# Associations between occupation and heavy alcohol consumption in UK adults aged 40–69 years: a cross-sectional study using the UK Biobank

**DOI:** 10.1186/s12889-021-10208-x

**Published:** 2021-02-24

**Authors:** Andrew Thompson, Munir Pirmohamed

**Affiliations:** 1 Wolfson Centre for Personalised Medicine, Molecular & Clinical Pharmacology, University of Liverpool, Liverpool, UK; 2 MRC Centre for Drug Safety Science, Molecular & Clinical Pharmacology, University of Liverpool, Liverpool, UK; 3 Liverpool Centre for Alcohol Research, University of Liverpool, Liverpool, UK

**Keywords:** Alcohol use, Occupation, UK biobank, Cross sectional study

## Abstract

**Background:**

Understanding the relationship between occupation and alcohol use offers opportunities to provide health promotion programmes based on evidence of need. We aimed to determine associations between occupation and heavy alcohol consumption in working individuals aged 40–69 years.

**Methods:**

A cross-sectional study was conducted using 100,817 people from the UK Biobank: 17,907 participants categorised as heavy drinkers, defined as > 35 units/week for women and > 50 units/week for men, and 82,910 drinking controls. Prevalence ratios (PRs) and 95% CIs were calculated for gender-specific heavy drinking in 353 occupations using Standard Occupational Classification, V.2000.

**Results:**

Seventy-seven occupations were associated with level of alcohol consumption in drinkers. The largest ratios for heavy drinkers were observed for publicans and managers of licenced premises (PR = 2.81, 95%CI 2.52–3.14); industrial cleaning process occupations (PR = 2.09, 1.33–3.28); and plasterers (PR = 2.07, 1.66–2.59). Clergy (PR = 0.20, 0.13–0.32); physicists, geologists and meteorologists (PR = 0.40, 0.25–0.65); and medical practitioners (PR = 0.40, 0.32–0.50) were least likely to be heavy drinkers. There was evidence of gender-specific outcomes with the proportion of jobs associated with heavy drinking accounted for by skilled trade occupations being 0.44 for males and 0.05 for females, and 0.10 for males and 0.40 for females when considering managers and senior officials.

**Conclusions:**

In the largest study of its kind, we found evidence for associations between a wider variety of occupations and the risk of heavy alcohol consumption than identified previously, particularly in females, although causality cannot be assumed. These results help determine which jobs and broader employment sectors may benefit most from prevention programmes.

**Supplementary Information:**

The online version contains supplementary material available at 10.1186/s12889-021-10208-x.

## Background

Alcohol consumption and its associated consequences remain a major public health challenge, and determining the factors that contribute to alcohol consumption, especially very high levels, is important for deciding where to target intervention resources. The estimated cost to the UK economy of lost productivity due to alcohol was £7.3 billion in 2009–2010 [1], equivalent to £9.2 billion in 2018. Raising productivity is one of the UK Government’s key priorities and is central to the UK’s Industrial Strategy [2]. Understanding the relationship between occupation and alcohol use offers opportunities to provide efficient and economical health promotion programmes based on evidence of need.

From an individual perspective, alcohol use increases the risk of physical and mental harm which impact health and can lead to undesirable labour market outcomes such as loss of personal income, injury, and termination of employment [3]. Job loss can also lead to worse outcomes in alcohol use through increased consumption and increased risk of morbidity and death [4]. From a business perspective, alcohol has been linked to decreased productivity, absenteeism/presenteeism, neglect of co-workers safety, and antisocial behaviours in the workplace [5, 6]. For example, high risk drinkers in Australia were approximately 22 times more likely to be absent from work due to their alcohol use compared to low risk drinkers [7]. Employers often sustain many of the gross financial consequences associated with alcohol misuse, and the impact is disproportionately large for small businesses. Data from 2805 employed adults in the US estimates that the prevalence of workforce impairment due to alcohol use is 15%, with variation across different occupation sectors [8]. Furthermore, a recent survey in the UK suggested that working hungover or under the influence of alcohol costs the UK economy between £1.2 billion and £1.4 billion a year; approximately £900 million more than previous estimates [9].

National level data has been used to good effect in several countries to observe links between job type and alcohol-related outcomes. Evidence from the Finnish care register demonstrated that manual workers in craft, construction and service industries were at greatest risk of hospitalisation or death primarily caused by alcohol [10]. Household survey data from the US found an association between higher rates of alcohol use disorders and employment in transport and construction industries when analyzing 104 occupations [11]. Register-based analysis from Sweden also highlighted increased relative risk of alcohol use disorder diagnosis and mortality due to alcohol in several jobs that were mainly manual [12]. Most of these studies have focused on morbidity and/or mortality, but evidence for how different jobs affect alcohol consumption itself is scarce, including in the UK where data has focused on mortality outcomes [13]. This is important as alcohol is a contributing factor in many conditions, not only those that are wholly attributable to alcohol (e.g. alcohol-related liver disease). Furthermore, investigations using alcohol consumption phenotypes are of specific interest to the field as they present an opportunity for preventive interventions in targeted groups.

Our aim in this cross-sectional study was to determine if certain occupations are associated with increased rates of heavy alcohol consumption in working individuals who drink and are aged 40–69 years from UK Biobank (http://www.ukbiobank.ac.uk/). We perform analysis across 353 occupations, investigate whether associations are gender dependent, and estimate how much variance in alcohol consumption status is explained by self-reported job.

## Methods

### Study population

The UK Biobank is a large population cohort of ~ 502,000 individuals from the UK aged 40–69 years at the time of recruitment. Individuals with contact information available via National Health Service central registers who met the age and distance from recruitment centre criteria were invited by letter to join the study (~ 9 million people). Baseline assessment was undertaken between 2006 and 2010 at one of 22 centres across the UK; 89% were recruited from 17 centres in England, 7% from two in Scotland, and 4% from three in Wales. Each participant completed a comprehensive demographic, lifestyle and health questionnaire, underwent clinical measures, provided biological samples (i.e. blood, urine and saliva), and agreed to have their health records accessed [14].

Ethical approval for UK Biobank was gained from the Research Ethics Service (REC reference: 15/NW/0274), and written informed consent was obtained from all participants. The current study was conducted under approved UK Biobank data application number 15110.

### Phenotype definition

Questions from the UK Biobank baseline assessment were used to develop two study groups: heavy drinkers (cases) and drinkers not reaching criteria for cases (controls). Abstainers were not included due to uncertainty regarding reason for current abstinence (e.g. former heavy drinkers that were now abstinent) and the aim of understanding behaviour in drinkers. All participants that indicated they consumed alcohol were asked to quantify their intake per week or per month using standard drink sizes (e.g. “In an average WEEK, how many glasses of RED wine would you drink? [There are six glasses in an average bottle]”); pictures accompanied these questions to provide visual representation of each measure. We then applied a standardised number of UK alcohol units to each drink to enable an estimated number of units per week to be calculated. Gender-specific heavy drinking was then defined as > 35 units/week for women and > 50 units/week for men. The cut-offs are based on published evidence stating that drinking at these levels puts individuals at high risk of physical and/or mental harm [16]. Controls were individuals that were not current abstainers from alcohol (i.e. ≥ 1 unit per week) but did not reach the gender-specific criteria for heavy drinking and were drinking at similar levels to 10 years previous. The final element of the control group criteria was implemented to reduce risk of movement between study groups (i.e. increase the likelihood of consistent drinking levels over time and reduce random variation).

### Employment and job code

Employment status was available for > 99% of UK Biobank participants. Verbal interviews were conducted with those that indicated their status as being “In paid employment or self-employed”. Trained interviewers subsequently coded the participants’ job using the four-digit Standard Occupational Classification (SOC), V.2000. This coding system operates a hierarchal tree structure with the four-digit SOC corresponding to one of 353 occupations. A deduced job code was utilised for those where “Other job” was entered. The certainty of these deduced codes was assessed by Cascot confidence score [15], with the highest score for each participant being retained. This study utilised a Cascot confidence score cut-off of ≥50 for inclusion of deduced jobs in the final dataset; participants were entered into the reference group where the score was below the cut-off.

### Statistical analysis

In cross-sectional studies with binary outcomes, the association between exposure and outcome is estimated by means of prevalence ratios. Here, Poisson regression models with robust standard errors were performed as an alternative to logistic regression to examine the association of case/control alcohol consumption status with current occupation, where all employed participants not working in each specific job were used as the reference group. This approach allows the direct estimation of prevalence ratios (PRs) through the exponential function of the Poisson model coefficient and associated 95% confidence intervals (95% CI) to be calculated for each job whilst negating convergence problems of binomial models when the prevalence of the outcome is high or if any of the covariates are continuous [17, 18]. A PR > 1 represents a higher likelihood of case status when employed in the investigated occupation; a PR < 1 represents a higher likelihood of control status.

The base model included all participants and age, sex and recruitment centre (*n* = 22) as covariates. Additional covariates (index of multiple deprivation, disability status, and ethnicity) were added to the model and a change in effect size of > 10% was considered evidence for including the variable to account for potential bias. The final model was adjusted for age, gender, recruitment centre and index of multiple deprivation, based on Townsend score [19]. Participants were subsequently stratified by gender and the models rerun to explore whether there was evidence of differential outcomes between males and females. All jobs with counts < 5 in either cases or controls were excluded from the results in both the combined and stratified analyses. A false discovery rate correction was applied to account for multiple comparisons and risk of type I errors, and all reported *p*-values associated with PRs are the corrected versions. Where occupations reached statistical significance, we explored trends with duration of employment (10-year categories) as a proxy for exposure-response using a chi-squared test for trend in proportions. Finally, we estimated the amount of variance explained in case-control status by occupation using McFadden’s R-squared. All analysis was performed using R (V3.5.0 or higher).

#### Sensitivity analyses

1. Increase the cut-off for counts in either controls or cases from < 5 to < 50.
2. Aggregate occupations to two-digit SOC, V.2000.

### Role of the funding source

The funder did not engage in the design and conduct of the study; collection, management, analysis, and interpretation of the data; or preparation, review, and approval of the manuscript.

## Results

### Cohort characteristics

**Fig. 1:**
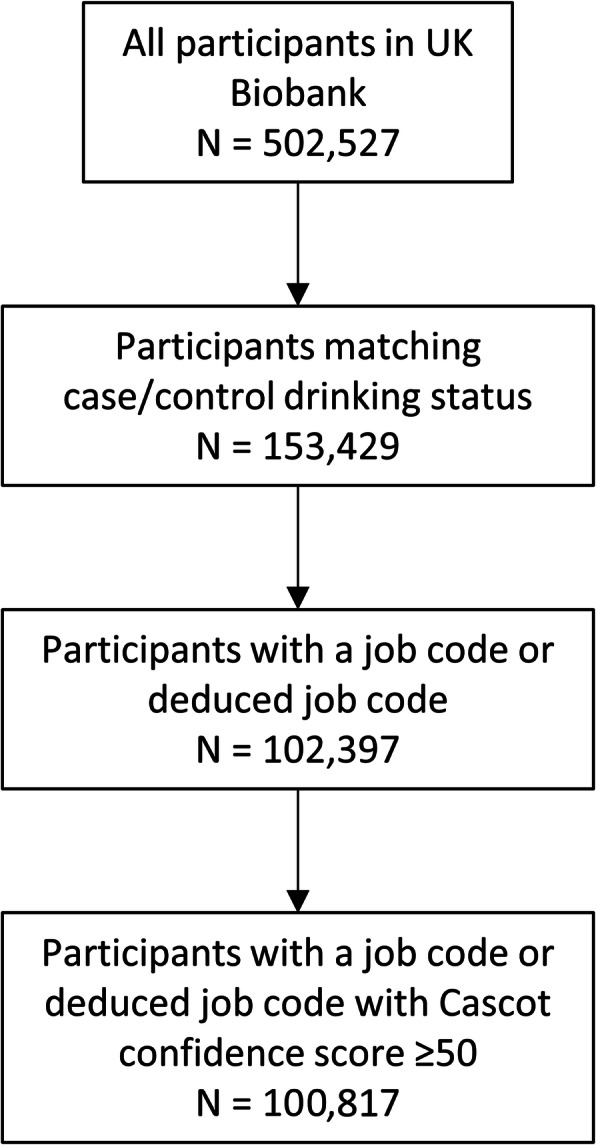
Determination of cohort size

There were 100,817 UK Biobank participants included in this study (Fig. 1); 46% were females and the average age was 55 years (SD = 8). The study sample deviated significantly from the rest of the UK Biobank population in terms of basic characteristics, with evidence of a greater portion of males (54% vs. 43%; χ^2^ = 3678.1, *p* < 0.0001) and younger mean age (55 vs. 57 years; *p* < 0.0001). The difference in age is likely a result of retired participants, who are generally older, not matching study eligibility criteria (i.e. not currently employed). The difference in gender is likely a result of a greater proportion of males being active in the UK workforce. There were 17,907 participants categorised as heavy drinkers (i.e. cases), of which 5154 (28.8%) were females. Cases were more likely to be males and younger than controls (both *P* < 0.05, data not shown).

### Main analysis

Thirty-six of the 353 jobs analysed were excluded because of counts < 5 in at least one group. There was evidence, following correction for multiple comparisons, of 77 occupations having an association with alcohol consumption status in drinkers (Table S1), with 51 having a higher ratio of heavy drinkers (Table 1). The largest effect sizes for being a case were observed for publicans and managers of licenced premises (PR = 2.81, 95%CI 2.52–3.14); industrial cleaning process occupations (PR = 2.09, 1.33–3.28); plasterers (PR = 2.07, 1.66–2.59); and sport and leisure assistants (PR = 2.07, 1.45–2.97). Jobs classified under skilled trade occupations (*n* = 19) had the highest number of associations with heavy drinking.

**Table 1:** Occupations associated with increased risk of heavy alcohol consumption in the entire cohort

Job	Job code	Controls in job / not in job	Cases in job / not in job	RP	LCL	UCL	P-FDR
PUBLICANS AND MANAGERS OF LICENSED PREMISES	1224	68/82842	123/17784	2.81	2.52	3.14	7.51E-73
INDUSTRIAL CLEANING PROCESS OCCUPATIONS	9132	9/82901	10/17897	2.09	1.33	3.28	7.86E-03
PLASTERERS	5321	32/82878	40/17867	2.07	1.66	2.59	3.62E-09
SPORTS AND LEISURE ASSISTANTS	6211	37/82873	19/17888	2.07	1.45	2.97	6.31E-04
BAR STAFF	9225	48/82862	36/17871	2.06	1.62	2.63	8.99E-08
REFUSE AND SALVAGE OCCUPATIONS	9235	11/82899	16/17891	2.05	1.47	2.87	2.49E-04
WEIGHERS, GRADERS, SORTERS	8134	7/82903	5/17902	2.04	1.27	3.29	1.72E-02
AUTO ELECTRICIANS	5233	12/82898	10/17897	2.04	1.26	3.30	1.90E-02
ROOFERS, ROOF TILERS AND SLATERS	5313	25/82885	34/17873	1.90	1.55	2.33	1.23E-08
VEHICLE BODY BUILDERS AND REPAIRERS	5232	22/82888	20/17887	1.84	1.32	2.56	2.64E-03
GLAZIERS, WINDOW FABRICATORS AND FITTERS	5316	35/82875	33/17874	1.83	1.45	2.31	5.29E-06
PIPE FITTERS	5216	23/82887	27/17880	1.82	1.39	2.38	1.16E-04
BRICKLAYERS, MASONS	5312	82/82828	76/17831	1.80	1.52	2.14	3.03E-10
FLOORERS AND WALL TILERS	5322	45/82865	40/17867	1.79	1.44	2.22	2.26E-06
STEEL ERECTORS	5311	19/82891	18/17889	1.78	1.28	2.46	3.94E-03
BEAUTICIANS AND RELATED OCCUPATIONS	6222	60/82850	16/17891	1.71	1.13	2.61	4.93E-02
CONSTRUCTION TRADES NEC	5319	408/82502	261/17646	1.66	1.51	1.82	2.34E-24
ASSEMBLERS (VEHICLES AND METAL GOODS)	8132	46/82864	33/17874	1.64	1.27	2.11	1.09E-03
METAL PLATE WORKERS, SHIPWRIGHTS, RIVETERS	5214	22/82888	15/17892	1.62	1.12	2.33	4.21E-02
PLUMBERS, HEATING AND VENTILATING ENGINEERS	5314	338/82572	226/17681	1.62	1.46	1.79	3.64E-19
CHEMICAL AND RELATED PROCESS OPERATIVES	8114	68/82842	50/17857	1.59	1.30	1.94	6.70E-05
SCAFFOLDERS, STAGERS, RIGGERS	8141	25/82885	23/17884	1.59	1.20	2.09	6.81E-03
ROAD CONSTRUCTION OPERATIVES	8142	33/82877	25/17882	1.58	1.20	2.08	7.80E-03
CONSTRUCTION OPERATIVES NEC	8149	99/82811	65/17842	1.56	1.30	1.88	2.77E-05
FORK-LIFT TRUCK DRIVERS	8222	70/82840	54/17853	1.56	1.29	1.88	4.99E-05
LABOURERS IN BUILDING AND WOODWORKING TRADES	9121	124/82786	82/17825	1.56	1.32	1.84	3.41E-06
PAINTERS AND DECORATORS	5323	198/82712	114/17793	1.52	1.32	1.75	1.24E-07
WELDING TRADES	5215	88/82822	60/17847	1.50	1.24	1.80	1.77E-04
SHOPKEEPERS AND WHOLESALE/RETAIL DEALERS	1234	357/82553	132/17775	1.48	1.29	1.71	9.81E-07
PRINTERS	5422	61/82849	37/17870	1.48	1.16	1.89	8.29E-03
CARPENTERS AND JOINERS	5315	388/82522	224/17683	1.47	1.32	1.63	1.77E-11
FOOD, DRINK AND TOBACCO PROCESS OPERATIVES	8111	86/82824	45/17862	1.47	1.16	1.85	8.29E-03
WINDOW CLEANERS	9231	35/82875	25/17882	1.46	1.09	1.97	4.85E-02
RESTAURANT AND CATERING MANAGERS	1223	195/82715	74/17833	1.43	1.18	1.73	2.33E-03
CARETAKERS	6232	156/82754	90/17817	1.42	1.21	1.66	1.28E-04
OTHER GOODS HANDLING AND STORAGE OCCUPATIONS NEC	9149	328/82582	197/17710	1.40	1.26	1.57	1.81E-08
COMPANY SECRETARIES	4214	223/82687	47/17860	1.39	1.08	1.80	4.93E-02
CUSTOMER CARE MANAGERS	1142	173/82737	53/17854	1.37	1.09	1.73	3.35E-02
ELECTRICIANS, ELECTRICAL FITTERS	5241	650/82260	305/17602	1.36	1.24	1.49	1.41E-09
LABOURERS IN PROCESS AND PLANT OPERATIONS NEC	9139	196/82714	99/17808	1.34	1.15	1.57	1.88E-03
METAL WORKING PRODUCTION AND MAINTENANCE FITTERS	5223	596/82314	283/17624	1.32	1.20	1.46	1.70E-07
POSTAL WORKERS, MAIL SORTERS, MESSENGERS, COURIERS	9211	303/82607	147/17760	1.32	1.16	1.50	2.12E-04
BROKERS	3532	168/82742	63/17844	1.31	1.06	1.62	4.93E-02
TRANSPORT AND DISTRIBUTION MANAGERS	1161	214/82696	88/17819	1.31	1.10	1.56	1.63E-02
MANAGERS IN CONSTRUCTION	1122	617/82293	241/17666	1.28	1.15	1.42	8.13E-05
CHEFS, COOKS	5434	314/82596	99/17808	1.27	1.07	1.50	2.69E-02
HEAVY GOODS VEHICLE DRIVERS	8211	423/82487	213/17694	1.27	1.14	1.41	2.03E-04
VAN DRIVERS	8212	351/82559	156/17751	1.25	1.10	1.43	5.99E-03
CARE ASSISTANTS AND HOME CARERS	6115	725/82185	169/17738	1.24	1.09	1.42	7.23E-03
OFFICE MANAGERS	1152	879/82031	194/17713	1.24	1.09	1.41	5.91E-03
SALES REPRESENTATIVES	3542	644/82266	189/17718	1.18	1.04	1.34	3.80E-02

The occupations with the lowest ratio of heavy drinkers were: Clergy (PR = 0.20, 0.13–0.32); physicists; geologists and meteorologists (PR = 0.40, 0.25–0.65); medical practitioners (PR = 0.40, 0.32–0.50); and school secretaries (PR = 0.45, 0.28–0.71) (Table 2). The majority (17 of 26; 65%) of jobs associated with lower likelihood of being cases were broadly categorised as professional occupations under SOC. We then examined the occupations by duration of employment. After correction for multiple testing, 30 occupations associated with an increased risk of heavy drinking showed evidence of a trend. However, the direction of these trends was mixed (Table S2). Sixteen occupations associated with the lowest ratio of heavy drinkers demonstrated a trend with duration of employment, the majority (*n* = 11) showing a positive trend; i.e. greater time employed in these occupations was associated with increasing rates of lower heavy drinking (Table S3).

**Table 2:** Occupations associated with decreased risk of heavy alcohol consumption in the entire cohort

Job	Job code	Controls in job / not in job	Cases in job / not in job	RP	LCL	UCL	P-FDR
ACCOUNTS AND WAGES CLERKS, BOOK-KEEPERS, OTHER FINANCIAL CLERKS	4122	2209/80701	327/17580	0.87	0.78	0.96	2.93E-02
NURSES	3211	2121/80789	241/17666	0.82	0.72	0.92	5.99E-03
INFORMATION AND COMMUNICATION TECHNOLOGY MANAGERS	1136	1224/81686	236/17671	0.81	0.72	0.91	3.12E-03
CHARTERED AND CERTIFIED ACCOUNTANTS	2421	821/82089	139/17768	0.81	0.69	0.94	3.12E-02
EDUCATIONAL ASSISTANTS	6124	1075/81835	125/17782	0.81	0.69	0.95	4.40E-02
SECONDARY EDUCATION TEACHING PROFESSIONALS	2314	2678/80232	379/17528	0.80	0.73	0.88	5.92E-05
TEACHING PROFESSIONALS NEC	2319	854/82056	108/17799	0.78	0.66	0.93	3.12E-02
IT STRATEGY AND PLANNING PROFESSIONALS	2131	802/82108	147/17760	0.76	0.66	0.89	3.07E-03
MECHANICAL ENGINEERS	2122	504/82406	94/17813	0.75	0.62	0.90	1.21E-02
SOFTWARE PROFESSIONALS	2132	1041/81869	190/17717	0.72	0.63	0.82	1.91E-05
LIBRARIANS	2451	354/82556	35/17872	0.65	0.47	0.89	3.35E-02
RESEARCH AND DEVELOPMENT MANAGERS	1137	237/82673	30/17877	0.64	0.46	0.90	4.21E-02
CIVIL ENGINEERS	2121	624/82286	92/17815	0.62	0.51	0.75	1.36E-05
DESIGN AND DEVELOPMENT ENGINEERS	2126	213/82697	31/17876	0.62	0.45	0.85	1.72E-02
HIGHER EDUCATION TEACHING PROFESSIONALS	2311	1856/81054	239/17668	0.62	0.55	0.70	1.88E-13
LABORATORY TECHNICIANS	3111	314/82596	34/17873	0.61	0.45	0.83	1.06E-02
PENSIONS AND INSURANCE CLERKS	4132	212/82698	20/17887	0.54	0.36	0.81	1.60E-02
PRIMARY AND NURSERY EDUCATION TEACHING PROFESSIONALS	2315	2572/80338	179/17728	0.53	0.46	0.61	2.94E-16
ARCHITECTS	2431	435/82475	53/17854	0.52	0.40	0.67	6.99E-06
PHYSIOTHERAPISTS	3221	300/82610	19/17888	0.52	0.33	0.80	1.67E-02
TOWN PLANNERS	2432	127/82783	14/17893	0.50	0.31	0.83	3.46E-02
BIOLOGICAL SCIENTISTS AND BIOCHEMISTS	2112	578/82332	49/17858	0.49	0.37	0.64	2.67E-06
SCHOOL SECRETARIES	4213	335/82575	17/17890	0.45	0.28	0.71	4.43E-03
MEDICAL PRACTITIONERS	2211	1074/81836	80/17827	0.40	0.32	0.50	2.48E-15
PHYSICISTS, GEOLOGISTS AND METEOROLOGISTS	2113	183/82727	15/17892	0.40	0.25	0.65	1.86E-03
CLERGY	2444	374/82536	18/17889	0.20	0.13	0.32	2.36E-10

### Gender heterogeneity

**Fig. 2:**
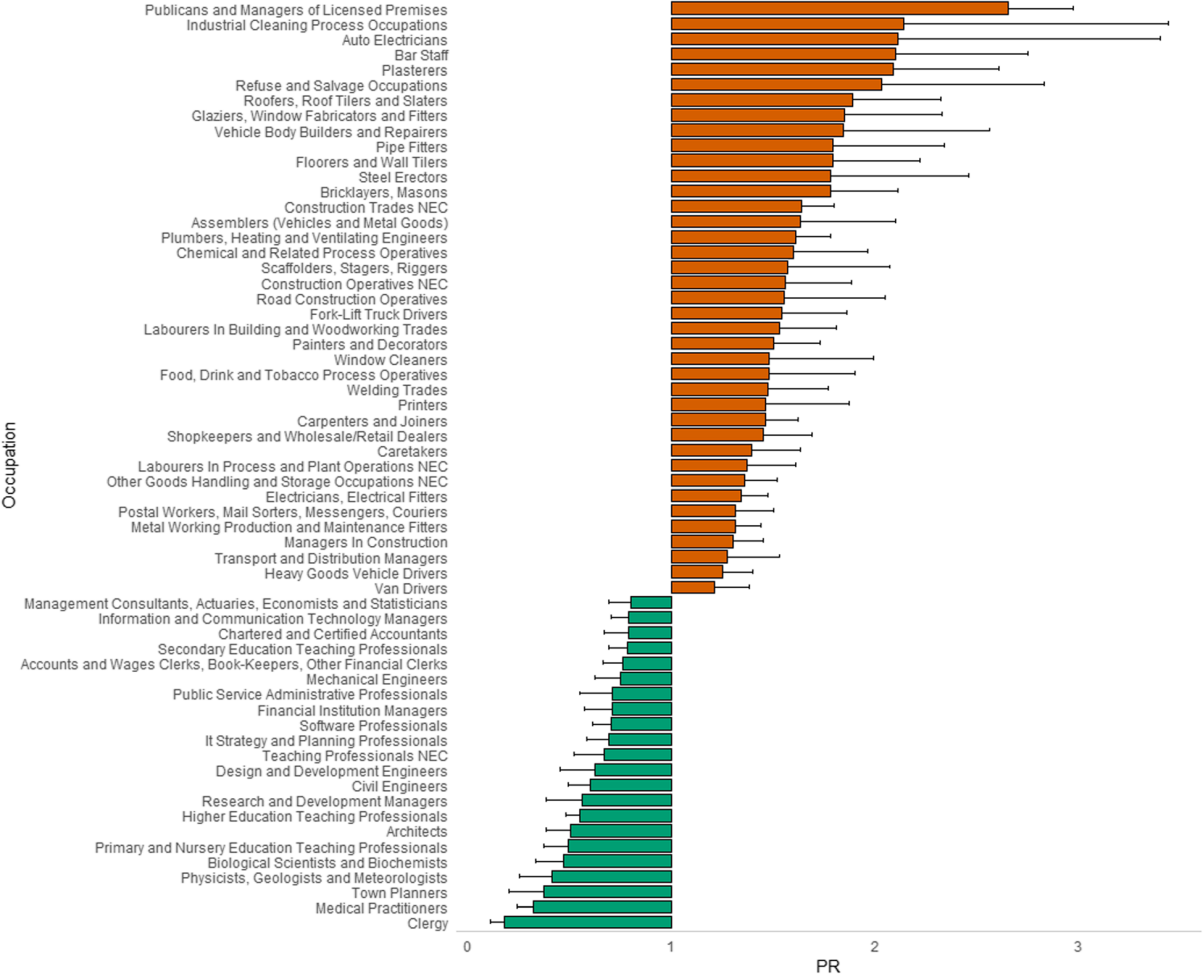
Prevalence ratios and associated single direction 95% confidence intervals for occupations obtaining post multiple testing correction significance in males

**Fig. 3:**
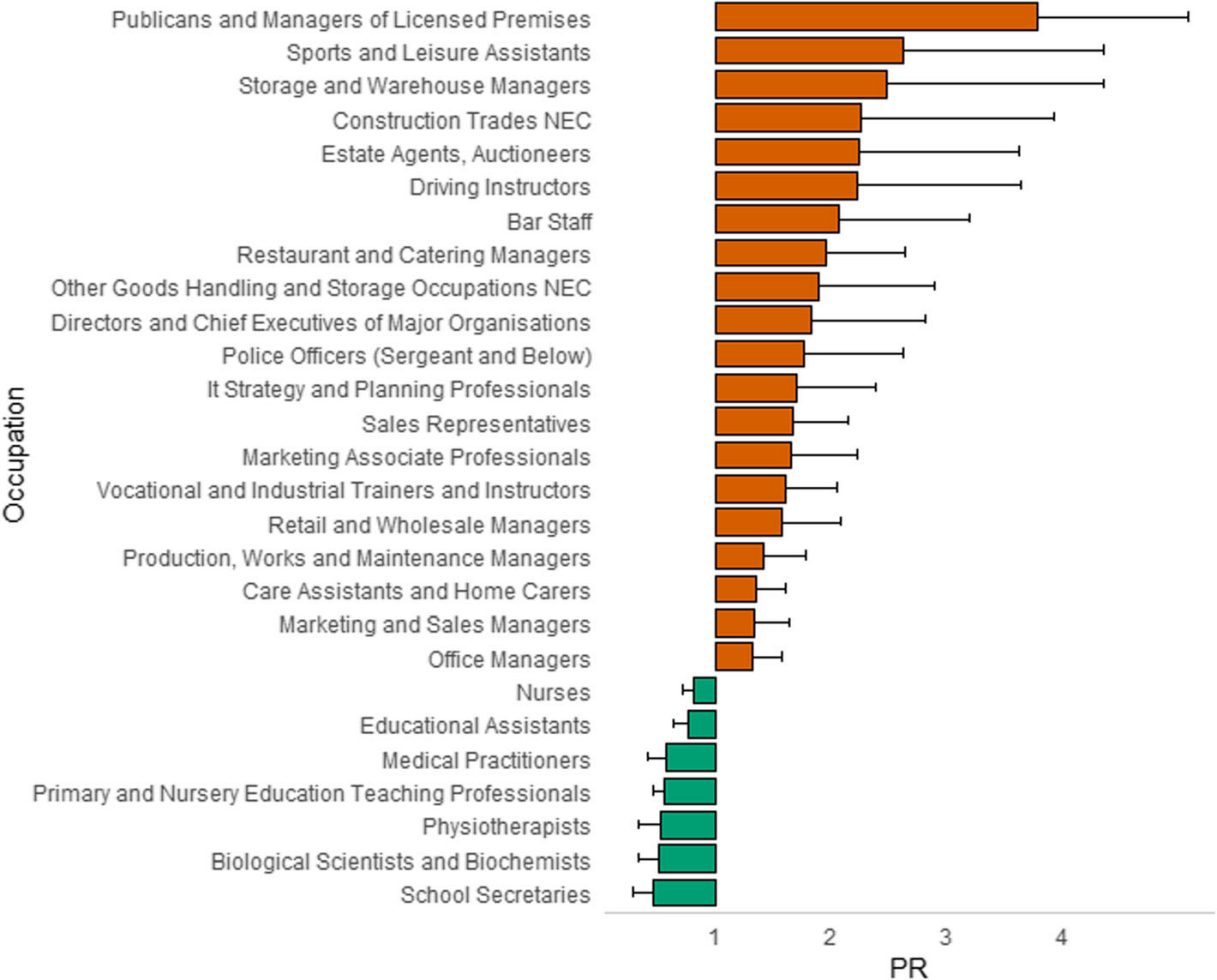
Prevalence ratios and associated single direction 95% confidence intervals for occupations obtaining post multiple testing correction significance in females

The number of occupations retained in the analysis when stratified by gender decreased to 279 for males and 170 for females. Evidence for an association was observed for 61 occupations in males (Table S4 and Fig. 2) and 27 in females (Table S5 and Fig. 3). Publicans and managers of licenced premises remained the job with the strongest association for heavy drinking in both genders, although the ratio was larger for females (PR = 3.79, 2.82–5.09) than males (PR = 2.65, 2.36–2.97). The other occupations with a PR > 2 for males were: industrial cleaning process occupations (PR = 2.14, 1.33–3.44); auto electricians (PR = 2.11, 1.31–3.40); bar staff (PR = 2.10, 1.60–2.75); plasterers (PR = 2.09, 1.68–2.61); and refuse and salvage occupations (PR = 2.03, 1.45–2.83). Skilled trade occupations remained the broad classification with most associations for heavy drinking in males. There were nine occupations with a PR > 2 for females including storage and warehouse managers (PR = 2.48, 1.41–4.37); estate agents’ auctioneers (PR = 2.24, 1.38–3.63); driving instructors (PR = 2.22, 1.35–3.64); and, bar staff (PR = 2.07, 1.35–3.19). Managers and senior officials, more specifically corporate managers, had most associations for heavy drinking in females.

The occupations with the lowest PR remained clergy (PR = 0.18, 0.11–0.30) for men and school secretaries for women (PR = 0.46, 0.29–0.74). Other occupations with the lowest ratio of heavy drinkers for males included medical practitioners (PR = 0.32, 0.24–0.42) and town planners (PR = 0.37, 0.20–0.69). For females, it included biological scientists and biochemists (PR = 0.51, 0.33–0.79) and physiotherapists (PR = 0.53, 0.33–0.84). The lower number of occupations reaching statistical significance prohibited meaningful interpretation of broad categories for females. For males, however, the majority of jobs associated with lower likelihood of being cases was consistent with the main analysis (i.e. professional occupations).

When directly comparing males and females, there was evidence of gender-specific associations being dependent on job group. Indeed, the proportion of total jobs associated with heavy drinking accounted for by skilled trade occupations was 0.44 for males and 0.05 for females, and 0.10 for males and 0.40 for females when considering managers and senior officials. Professional occupations had the lowest ratio of heavy drinkers for both sexes; although as above, interpretation is limited in females because of low numbers.

### Variance explained by occupation

The amount of variance explained when using all 353 occupations and only those in the main analysis was 5%. This reduced to 4.2% for males and 2.1% females when occupations were stratified by gender.

### Sensitivity analysis

When increasing the cut-off for counts in either controls or cases from < 5 to < 50, the number of occupations remaining for analysis reduced to 103 in the main cohort and to 70 and 27 in the analysis for males and females, respectively. This approach provides findings that are potentially more robust, although causation can still not be assumed. Publicans and managers of licenced premises remained the job with the highest ratio for heavy drinkers in the main cohort (Table S6), but the occupation was dropped from both gender-specific analyses. Medical practitioners had the lowest ratio of heavy drinkers in the main cohort. In males, bricklayers had the highest ratio of heavy drinkers and higher education teaching professionals had the lowest (Table S7). In females, vocational and industrial trainers and instructors had the highest ratio of heavy drinkers and primary and nursery education teaching professionals had the lowest (Table S8).

Aggregating the occupation data to two-digit SOC, V.2000 exhibits support for the main findings. Ninety-two percent of occupations associated with higher drinking and 86% of occupations associated with lower drinking in the main analysis, derive from two-digit classifications that demonstrate consistent direction of effect (e.g. most four-digit SOCs associated with heavy drinking derive from a two-digit SOC that is associated with heavy drinking) (Table S9).

## Discussion

In the largest such study to date, our aim was to identify whether certain occupations are associated with being a heavy alcohol drinker in 40–69 year olds. Our results show a wider variety of jobs are associated with heavy alcohol drinking than previously identified, whilst other occupations had lower ratios of heavy drinkers. There was also evidence that gender was a moderating factor in some circumstances but the effect of duration in current employment was varied within and between alcohol drinking groups. Identifying 17,907 cases means that our study has a higher number of individuals with the risk factor (e.g. drinking status) or outcome (e.g. alcohol-related death) of interest than other studies using occupation as their independent variable. Furthermore, the use of SOC allows us to match the largest study [13] in terms of occupations analysed with 353, the next closest being 104 [11]. Our data is consistent with previous findings, but because of the size of population studied, we have identified novel occupation-related associations with alcohol intake, providing important insights for preventive public health interventions.

We found robust evidence that publicans and managers of licenced premises were more likely to be heavy drinkers. This is complemented by findings since the 1890s where those routinely and directly working with alcohol, including publicans and bar staff, consistently demonstrate the highest rates of alcohol-related mortality [13, 20]. The same is observed at the opposite end of the spectrum where members of the clergy, identified as a protective occupation in our analysis, have consistently shown low rates of alcohol-related mortality. These contrary occupation-specific outcomes highlight how occupational environment, which may include specific societal and religious beliefs, influences relationships with alcohol.

One of the most consistent findings across our data was that occupations considered as ‘skilled trade’ had high ratios of heavy drinkers. Although this effect appears to be almost entirely driven by males who make up the majority (92.3% in 2008 [21]) of those working in this job group. Concordant data from Finland shows men working in construction and craft jobs were generally at the highest risk of alcohol-induced morbidity and mortality [10]. Similar results have been reported in Sweden [12] and the US [11, 22] for construction and mining, suggesting this effect is consistent across Western nations. There is also evidence from mortality data in England that those in skilled trade occupations are at increased risk of suicide, with this job group accounting for 29% of suicides in working males [23]. Whether a link between occupation, mental health, alcohol consumption and self-harm/suicide exists remains to be elucidated. For females, ‘managers’ as a broad category demonstrated the highest proportion of occupations with significant PRs for heavy drinking. This might be linked to job strain [24] or long working hours [25], although there is strong evidence from a multi-national study that women who are highly educated, a factor positively associated with senior employment, are more likely to be heavy drinkers [26].

Workplace health management has received growing interest as both an employer’s duty of care for providing a safe working environment and to benefit productivity. Workplace interventions are one mechanism of health promotion that have the potential to access specific groups who are often hard to reach, and have the advantage of potentially increased exposure given the large amount of time spent at work [27]. Several studies that integrated alcohol interventions into health promotion programmes using a combination of educational, counselling and brief intervention strategies reported promising results [28, 29], whilst others have had null effects [30]. A recent web-based alcohol intervention was shown to have positive outcomes in moderate drinkers [31], whilst also negating the potential fears of stigmatisation amongst co-workers [32]. Current evidence suggests that strategies which address organisational factors may be more effective than individually focused approaches. Indeed, workplace policy adjustment was recommend by Roche and colleagues to improve drinking culture through promoting social cohesion, modifying workplace drinking norms and tackling occupation-related factors that contribute to heavy drinking (e.g. demands, effort-reward deficits) [33]. A recent example of policy change in action is the introduction of a ban on alcohol consumption during core working hours by insurance market Lloyds of London. There is scope for workplace health management to be supported by the UK Government through the Industrial Strategy where ‘People’ is considered one of the ‘Five Foundations of Productivity’ [2].

There are other specific outcomes that warrant discussion. We found evidence that female police officers had a higher prevalence ratio for heavy drinking. The stressful nature of policing is a likely contributor. Indeed results from a study using members of a police service in Australia found that factors related to stress emerged as the most predictive factor for Alcohol Use Disorders Identification Test score [34]. There is also evidence that female officers take more sick days, impacting available deployable days across a force, which might be a result of stress and/or alcohol consumption [35]. Another specific outcome observed in females was that of a higher ratio of heavy drinking in driving instructors. Given that UK law deems the driving supervisor to be in control of the car and the general risk posed by motor vehicles, members of this group might be considered appropriate for screening and preventive non-specialist approaches such as brief interventions [36].

The variation in case control status explained by occupation alone was around 5%. This highlights the complex, interdependent determinants of alcohol consumption which includes genetic and non-genetic factors such gender [37, 38], age at first alcohol use [39], duration of poverty and involuntary unemployment [40], other lifestyle risk factors [41], and socio-economic status [42, 43]. Looking at individual factors, even when models are adjusted for other variables, might be an insufficient method for ensuring alcohol interventions target the correct groups. For example, evidence from Spain suggests that the unemployed have double the directly alcohol-attributable mortality compared to the employed [44], an outcome that is possibly related to socioeconomic differences in alcohol-related outcomes (i.e. the alcohol harm paradox) [45]. Thus, targeting specific occupations should be regarded as only one component of a multifaceted approach in reducing the harms associated with alcohol misuse.

### Strengths and limitations

The study is cross-sectional and therefore we are unable to infer causation. The case-control phenotypes are based on self-reported alcohol intake. It is well-documented that individuals under-report their alcohol consumption for a number of reasons. There is therefore a risk of cases being mislabeled as controls, alongside the granularity of the data being reduced in the categorical approach. However, the large sample size of > 100,000 helps provide greater precision in our estimates alongside good power to detect differences. We also applied a formal correction for multiple comparisons, which gives us further confidence that the results are robust. There is however a need to acknowledge the greater proportion of males, and slightly younger mean age in our sample, than the entire UK Biobank cohort, and that UK Biobank has certain selection biases towards a “healthy volunteer” population which means it is unlikely to completely represent the population workforce [46]. Indeed, we used classifications from the SOC to explore the proportions employed in each major occupation group between our UK Biobank subgroup and estimates from the Labour Force Survey in 2008 (mid-point in UK Biobank recruitment) [21] and found differences in group representation (Table S10). There were substantially more people in UK Biobank employed in professional occupations (27.6% vs. 12.8%) and less in occupations classed as Personal Service (4.6% vs. 8.3%), Sales and Customer Service (2.8% vs. 7.6%), Process Plan and Machine Operatives (4.2% vs. 7.1%), and Elementary (3.9% vs. 11.6%). Such deviations may also be explained by this analysis only including individuals aged between 40 and 69 years, and we caution against extrapolation outside this age group. We performed sensitivity analysis to account for concerns of low power by increasing the threshold of occupation exclusion from < 5 to < 50 counts in either cases or controls and by aggregating occupations to two-digit SOC, V.2000. Finally, the data were collected 2006–2010 which means we may have missed changes in drinking patterns and occupations in the intervening years, although evidence from the ONS suggests that, with the exception of managers / senior officials and professional occupations, the proportion employed in each broad occupation category is similar between 2008 and 2018 (Table S11).

## Conclusion

Overall, we found robust associations between occupations reported by the study participants and heavy alcohol consumption, but these are not evidence of causation. Jobs identified as skilled trades were most likely to be associated with heavy alcohol consumption. Those working in other industries, especially with links to alcohol, also demonstrate increased propensity for heavy drinking. There was also consistent evidence that workers in professional occupations were less likely to drink at high levels. Understanding which occupations, together with other factors, are associated with heavy alcohol consumption is important to ensure that resources for interventions are appropriately targeted to elicit maximum benefit. Workplace interventions and policies have the potential to act as prevention measures in occupations where heavy drinking is prevalent. Such effective measures are likely to benefit the individual, business and the wider economy through improved productivity.

## Supplementary Information

**Additional file 1.** Supplementary Tables S1-S11. All supplementary tables as listed in the main manuscript.

## Data Availability

Data available under license from the UK Biobank. Information detailing how to gain access to UK Biobank can be found at https://www.ukbiobank.ac.uk/.

## Abbreviations

PR: Prevalence ratios
SOC: Standard Occupational Classification
95% CI: 95% confidence intervals
SD: Standard deviation
