# Supplementary material for: Associations between occupation and heavy alcohol consumption in UK adults aged 40–69 years: a cross-sectional study using the UK Biobank
Source: BMC Public Health. 2021 Feb 24;21:190. doi: 10.1186/s12889-021-10208-x (PMC7903617; doi:10.1186/s12889-021-10208-x)
Supplement: Supplementary file 1 — Additional file 1. Supplementary Tables S1-S11. All supplementary tables as listed in the main manuscript. [file 12889_2021_10208_MOESM1_ESM.docx]

**Supplementary material**

**Associations between occupation and heavy alcohol consumption in UK adults aged 40-69 years: A cross-sectional study using the UK Biobank study**

***BMC Public Health***

Andrew Thompson^1,2,3^, Munir Pirmohamed^1,2,3^

^1^Wolfson Centre for Personalised Medicine, Molecular & Clinical Pharmacology, University of Liverpool, United Kingdom

^2^MRC Centre for Drug Safety Science, Molecular & Clinical Pharmacology, University of Liverpool, United Kingdom

^3^Liverpool Centre for Alcohol Research, University of Liverpool, United Kingdom

Corresponding author email: [andrew.thompson@liverpool.ac.uk](mailto:andrew.thompson@liverpool.ac.uk)

**Table S1: Outcomes for all occupations, ordered by PR**

| **Job** | **Job code** | **Controls in job / not in job** | **Cases in job / not in job** | **PR** | **LCL** | **UCL** | **P-FDR** |
| --- | --- | --- | --- | --- | --- | --- | --- |
| Publicans and Managers of Licensed Premises | 1224 | 68/82842 | 123/17784 | 2.81 | 2.52 | 3.14 | 7.51E-73 |
| Industrial Cleaning Process Occupations | 9132 | 9/82901 | 10/17897 | 2.09 | 1.33 | 3.28 | 7.86E-03 |
| Plasterers | 5321 | 32/82878 | 40/17867 | 2.07 | 1.66 | 2.59 | 3.62E-09 |
| Sports and Leisure Assistants | 6211 | 37/82873 | 19/17888 | 2.07 | 1.45 | 2.97 | 6.31E-04 |
| Bar Staff | 9225 | 48/82862 | 36/17871 | 2.06 | 1.62 | 2.63 | 8.99E-08 |
| Refuse and Salvage Occupations | 9235 | 11/82899 | 16/17891 | 2.05 | 1.47 | 2.87 | 2.49E-04 |
| Weighers, Graders, Sorters | 8134 | 7/82903 | 5/17902 | 2.04 | 1.27 | 3.29 | 1.72E-02 |
| Auto Electricians | 5233 | 12/82898 | 10/17897 | 2.04 | 1.26 | 3.30 | 1.90E-02 |
| Roofers, Roof Tilers and Slaters | 5313 | 25/82885 | 34/17873 | 1.90 | 1.55 | 2.33 | 1.23E-08 |
| Tyre, Exhaust and Windscreen Fitters | 8135 | 5/82905 | 5/17902 | 1.86 | 0.96 | 3.62 | 1.66E-01 |
| Vehicle Body Builders and Repairers | 5232 | 22/82888 | 20/17887 | 1.84 | 1.32 | 2.56 | 2.64E-03 |
| Glaziers, Window Fabricators and Fitters | 5316 | 35/82875 | 33/17874 | 1.83 | 1.45 | 2.31 | 5.29E-06 |
| Pipe Fitters | 5216 | 23/82887 | 27/17880 | 1.82 | 1.39 | 2.38 | 1.16E-04 |
| Bricklayers, Masons | 5312 | 82/82828 | 76/17831 | 1.80 | 1.52 | 2.14 | 3.03E-10 |
| Floorers and Wall Tilers | 5322 | 45/82865 | 40/17867 | 1.79 | 1.44 | 2.22 | 2.26E-06 |
| Coal Mine Operatives | 8122 | 12/82898 | 8/17899 | 1.78 | 1.07 | 2.95 | 8.80E-02 |
| Stevedores, Dockers and Slingers | 9141 | 8/82902 | 7/17900 | 1.78 | 1.02 | 3.11 | 1.24E-01 |
| Steel Erectors | 5311 | 19/82891 | 18/17889 | 1.78 | 1.28 | 2.46 | 3.94E-03 |
| Managers In Animal Husbandry, Forestry and Fishing NEC | 1219 | 21/82889 | 9/17898 | 1.76 | 1.02 | 3.05 | 1.24E-01 |
| Importers, Exporters | 3536 | 17/82893 | 8/17899 | 1.73 | 0.98 | 3.06 | 1.57E-01 |
| Beauticians and Related Occupations | 6222 | 60/82850 | 16/17891 | 1.71 | 1.13 | 2.61 | 4.93E-02 |
| Water and Sewerage Plant Operatives | 8126 | 9/82901 | 6/17901 | 1.66 | 0.92 | 2.99 | 2.02E-01 |
| Construction Trades NEC | 5319 | 408/82502 | 261/17646 | 1.66 | 1.51 | 1.82 | 2.34E-24 |
| Assemblers (Vehicles and Metal Goods) | 8132 | 46/82864 | 33/17874 | 1.64 | 1.27 | 2.11 | 1.09E-03 |
| Smiths and Forge Workers | 5211 | 10/82900 | 5/17902 | 1.63 | 0.83 | 3.20 | 2.90E-01 |
| Metal Plate Workers, Shipwrights, Riveters | 5214 | 22/82888 | 15/17892 | 1.62 | 1.12 | 2.33 | 4.21E-02 |
| Plumbers, Heating and Ventilating Engineers | 5314 | 338/82572 | 226/17681 | 1.62 | 1.46 | 1.79 | 3.64E-19 |
| Agricultural and Fishing Trades NEC | 5119 | 12/82898 | 7/17900 | 1.61 | 0.92 | 2.84 | 2.12E-01 |
| Officers In Armed Forces | 1171 | 15/82895 | 7/17900 | 1.61 | 0.84 | 3.09 | 2.87E-01 |
| Telephonists | 4141 | 43/82867 | 11/17896 | 1.60 | 0.98 | 2.62 | 1.57E-01 |
| Collector Salespersons and Credit Agents | 7121 | 26/82884 | 11/17896 | 1.59 | 1.01 | 2.50 | 1.25E-01 |
| Chemical and Related Process Operatives | 8114 | 68/82842 | 50/17857 | 1.59 | 1.30 | 1.94 | 6.70E-05 |
| Scaffolders, Stagers, Riggers | 8141 | 25/82885 | 23/17884 | 1.59 | 1.20 | 2.09 | 6.81E-03 |
| Metal Making and Treating Process Operatives | 8117 | 15/82895 | 14/17893 | 1.58 | 1.08 | 2.32 | 6.98E-02 |
| Road Construction Operatives | 8142 | 33/82877 | 25/17882 | 1.58 | 1.20 | 2.08 | 7.80E-03 |
| Energy Plant Operatives | 8124 | 17/82893 | 10/17897 | 1.57 | 0.98 | 2.53 | 1.60E-01 |
| Construction Operatives NEC | 8149 | 99/82811 | 65/17842 | 1.56 | 1.30 | 1.88 | 2.77E-05 |
| Fork-Lift Truck Drivers | 8222 | 70/82840 | 54/17853 | 1.56 | 1.29 | 1.88 | 4.99E-05 |
| Labourers In Building and Woodworking Trades | 9121 | 124/82786 | 82/17825 | 1.56 | 1.32 | 1.84 | 3.41E-06 |
| Painters and Decorators | 5323 | 198/82712 | 114/17793 | 1.52 | 1.32 | 1.75 | 1.24E-07 |
| Upholsterers | 5412 | 28/82882 | 11/17896 | 1.51 | 0.95 | 2.40 | 1.93E-01 |
| Estate Agents, Auctioneers | 3544 | 85/82825 | 26/17881 | 1.51 | 1.08 | 2.11 | 6.35E-02 |
| Air Travel Assistants | 6214 | 98/82812 | 28/17879 | 1.50 | 1.08 | 2.08 | 5.98E-02 |
| Welding Trades | 5215 | 88/82822 | 60/17847 | 1.50 | 1.24 | 1.80 | 1.77E-04 |
| Rail Transport Operatives | 8216 | 22/82888 | 14/17893 | 1.49 | 1.00 | 2.23 | 1.42E-01 |
| Glass and Ceramics Process Operatives | 8112 | 10/82900 | 6/17901 | 1.49 | 0.76 | 2.89 | 4.01E-01 |
| Shopkeepers and Wholesale/Retail Dealers | 1234 | 357/82553 | 132/17775 | 1.48 | 1.29 | 1.71 | 9.81E-07 |
| Printers | 5422 | 61/82849 | 37/17870 | 1.48 | 1.16 | 1.89 | 8.29E-03 |
| Crane Drivers | 8221 | 15/82895 | 10/17897 | 1.48 | 0.97 | 2.26 | 1.70E-01 |
| Travel and Tour Guides | 6213 | 63/82847 | 15/17892 | 1.48 | 0.93 | 2.34 | 2.12E-01 |
| Communication Operators | 4142 | 48/82862 | 17/17890 | 1.47 | 0.99 | 2.17 | 1.42E-01 |
| Carpenters and Joiners | 5315 | 388/82522 | 224/17683 | 1.47 | 1.32 | 1.63 | 1.77E-11 |
| Food, Drink and Tobacco Process Operatives | 8111 | 86/82824 | 45/17862 | 1.47 | 1.16 | 1.85 | 8.29E-03 |
| Window Cleaners | 9231 | 35/82875 | 25/17882 | 1.46 | 1.09 | 1.97 | 4.85E-02 |
| Animal Care Occupations NEC | 6139 | 36/82874 | 9/17898 | 1.46 | 0.82 | 2.59 | 3.49E-01 |
| Butchers, Meat Cutters | 5431 | 34/82876 | 18/17889 | 1.45 | 1.03 | 2.02 | 1.02E-01 |
| Security Managers | 1174 | 32/82878 | 14/17893 | 1.44 | 0.95 | 2.17 | 1.96E-01 |
| Restaurant and Catering Managers | 1223 | 195/82715 | 74/17833 | 1.43 | 1.18 | 1.73 | 2.33E-03 |
| Rail Construction and Maintenance Operatives | 8143 | 11/82899 | 7/17900 | 1.42 | 0.76 | 2.68 | 4.40E-01 |
| Garage Managers and Proprietors | 1232 | 59/82851 | 24/17883 | 1.42 | 1.01 | 1.99 | 1.24E-01 |
| Mobile Machine Drivers and Operatives NEC | 8229 | 33/82877 | 20/17887 | 1.42 | 1.01 | 1.99 | 1.24E-01 |
| Caretakers | 6232 | 156/82754 | 90/17817 | 1.42 | 1.21 | 1.66 | 1.28E-04 |
| Other Goods Handling and Storage Occupations NEC | 9149 | 328/82582 | 197/17710 | 1.40 | 1.26 | 1.57 | 1.81E-08 |
| Company Secretaries | 4214 | 223/82687 | 47/17860 | 1.39 | 1.08 | 1.80 | 4.93E-02 |
| Quarry Workers and Related Operatives | 8123 | 10/82900 | 6/17901 | 1.38 | 0.73 | 2.60 | 4.91E-01 |
| Sales Related Occupations NEC | 7129 | 127/82783 | 40/17867 | 1.38 | 1.06 | 1.80 | 6.75E-02 |
| Forestry Workers | 9112 | 11/82899 | 6/17901 | 1.37 | 0.73 | 2.60 | 5.03E-01 |
| Customer Care Managers | 1142 | 173/82737 | 53/17854 | 1.37 | 1.09 | 1.73 | 3.35E-02 |
| Hairdressing and Beauty Salon Managers and Proprietors | 1233 | 28/82882 | 6/17901 | 1.37 | 0.68 | 2.75 | 5.42E-01 |
| Labourers In Other Construction Trades NEC | 9129 | 23/82887 | 14/17893 | 1.37 | 0.91 | 2.06 | 2.66E-01 |
| Electricians, Electrical Fitters | 5241 | 650/82260 | 305/17602 | 1.36 | 1.24 | 1.49 | 1.41E-09 |
| Hotel and Accommodation Managers | 1221 | 144/82766 | 40/17867 | 1.36 | 1.03 | 1.78 | 9.10E-02 |
| TV, Video and Audio Engineers | 5244 | 32/82878 | 15/17892 | 1.35 | 0.88 | 2.08 | 3.16E-01 |
| Elementary Sales Occupations NEC | 9259 | 40/82870 | 12/17895 | 1.35 | 0.81 | 2.25 | 4.06E-01 |
| Hospital Porters | 9221 | 18/82892 | 11/17896 | 1.34 | 0.87 | 2.08 | 3.35E-01 |
| Labourers In Process and Plant Operations NEC | 9139 | 196/82714 | 99/17808 | 1.34 | 1.15 | 1.57 | 1.88E-03 |
| Glass and Ceramics Makers, Decorators and Finishers | 5491 | 51/82859 | 18/17889 | 1.33 | 0.90 | 1.97 | 2.84E-01 |
| Rail Travel Assistants | 6215 | 25/82885 | 14/17893 | 1.32 | 0.88 | 1.99 | 3.22E-01 |
| Metal Working Production and Maintenance Fitters | 5223 | 596/82314 | 283/17624 | 1.32 | 1.20 | 1.46 | 1.70E-07 |
| Postal Workers, Mail Sorters, Messengers, Couriers | 9211 | 303/82607 | 147/17760 | 1.32 | 1.16 | 1.50 | 2.12E-04 |
| Debt, Rent and Other Cash Collectors | 7122 | 65/82845 | 25/17882 | 1.32 | 0.94 | 1.86 | 2.42E-01 |
| Paper and Wood Machine Operatives | 8121 | 40/82870 | 24/17883 | 1.32 | 0.98 | 1.78 | 1.68E-01 |
| Leisure and Sports Managers | 1225 | 110/82800 | 37/17870 | 1.31 | 1.00 | 1.72 | 1.36E-01 |
| Sheet Metal Workers | 5213 | 46/82864 | 27/17880 | 1.31 | 0.98 | 1.76 | 1.69E-01 |
| Residential and Day Care Managers | 1185 | 124/82786 | 35/17872 | 1.31 | 0.98 | 1.75 | 1.66E-01 |
| Brokers | 3532 | 168/82742 | 63/17844 | 1.31 | 1.06 | 1.62 | 4.93E-02 |
| Transport and Distribution Managers | 1161 | 214/82696 | 88/17819 | 1.31 | 1.10 | 1.56 | 1.63E-02 |
| Tool Makers, Tool Fitters and Markers-Out | 5222 | 66/82844 | 27/17880 | 1.31 | 0.97 | 1.76 | 1.81E-01 |
| Managers In Mining and Energy | 1123 | 42/82868 | 16/17891 | 1.30 | 0.87 | 1.94 | 3.57E-01 |
| Marketing Associate Professionals | 3543 | 265/82645 | 67/17840 | 1.28 | 1.03 | 1.59 | 9.10E-02 |
| Managers In Construction | 1122 | 617/82293 | 241/17666 | 1.28 | 1.15 | 1.42 | 8.13E-05 |
| Senior Officers In Fire, Ambulance, Prison and Related Services | 1173 | 63/82847 | 25/17882 | 1.28 | 0.92 | 1.76 | 2.74E-01 |
| Elementary Security Occupations NEC | 9249 | 65/82845 | 23/17884 | 1.27 | 0.93 | 1.74 | 2.69E-01 |
| Statutory Examiners | 3566 | 34/82876 | 13/17894 | 1.27 | 0.82 | 1.97 | 4.51E-01 |
| Chefs, Cooks | 5434 | 314/82596 | 99/17808 | 1.27 | 1.07 | 1.50 | 2.69E-02 |
| Hand Craft Occupations NEC | 5499 | 24/82886 | 8/17899 | 1.27 | 0.74 | 2.18 | 5.48E-01 |
| Heavy Goods Vehicle Drivers | 8211 | 423/82487 | 213/17694 | 1.27 | 1.14 | 1.41 | 2.03E-04 |
| Lines Repairers and Cable Jointers | 5243 | 15/82895 | 8/17899 | 1.26 | 0.73 | 2.17 | 5.63E-01 |
| Elementary Personal Services Occupations NEC | 9229 | 20/82890 | 9/17898 | 1.25 | 0.73 | 2.16 | 5.67E-01 |
| Van Drivers | 8212 | 351/82559 | 156/17751 | 1.25 | 1.10 | 1.43 | 5.99E-03 |
| NCOs and Other Ranks | 3311 | 13/82897 | 6/17901 | 1.24 | 0.66 | 2.35 | 6.53E-01 |
| Care Assistants and Home Carers | 6115 | 725/82185 | 169/17738 | 1.24 | 1.09 | 1.42 | 7.23E-03 |
| Journalists, Newspaper and Periodical Editors | 3431 | 283/82627 | 78/17829 | 1.24 | 1.02 | 1.51 | 9.92E-02 |
| Assemblers (Electrical Products) | 8131 | 37/82873 | 14/17893 | 1.24 | 0.85 | 1.81 | 4.27E-01 |
| Office Managers | 1152 | 879/82031 | 194/17713 | 1.24 | 1.09 | 1.41 | 5.91E-03 |
| Telecommunications Engineers | 5242 | 176/82734 | 67/17840 | 1.23 | 1.01 | 1.50 | 1.13E-01 |
| Elementary Office Occupations NEC | 9219 | 126/82784 | 31/17876 | 1.23 | 0.91 | 1.66 | 3.21E-01 |
| Police Officers (Inspectors and Above) | 1172 | 69/82841 | 24/17883 | 1.23 | 0.87 | 1.73 | 4.04E-01 |
| Production and Process Engineers | 2127 | 70/82840 | 27/17880 | 1.21 | 0.88 | 1.67 | 3.94E-01 |
| Routine Inspectors and Testers | 8133 | 137/82773 | 48/17859 | 1.20 | 0.95 | 1.54 | 2.66E-01 |
| Stock Control Clerks | 4133 | 161/82749 | 53/17854 | 1.20 | 0.95 | 1.50 | 2.66E-01 |
| Musicians | 3415 | 143/82767 | 42/17865 | 1.19 | 0.93 | 1.53 | 3.09E-01 |
| Packers, Bottlers, Canners, Fillers | 9134 | 47/82863 | 17/17890 | 1.19 | 0.80 | 1.76 | 5.48E-01 |
| Retail and Wholesale Managers | 1163 | 538/82372 | 158/17749 | 1.18 | 1.03 | 1.36 | 6.01E-02 |
| Managers and Proprietors In Other Services NEC | 1239 | 531/82379 | 144/17763 | 1.18 | 1.03 | 1.36 | 7.01E-02 |
| Sales Representatives | 3542 | 644/82266 | 189/17718 | 1.18 | 1.04 | 1.34 | 3.80E-02 |
| Countryside and Park Rangers | 3552 | 19/82891 | 7/17900 | 1.18 | 0.65 | 2.16 | 7.28E-01 |
| Arts Officers, Producers and Directors | 3416 | 85/82825 | 25/17882 | 1.18 | 0.83 | 1.67 | 5.28E-01 |
| Telephone Salespersons | 7113 | 38/82872 | 10/17897 | 1.17 | 0.67 | 2.05 | 7.17E-01 |
| Taxi, Cab Drivers and Chauffeurs | 8214 | 255/82655 | 104/17803 | 1.17 | 1.00 | 1.38 | 1.50E-01 |
| Directors and Chief Executives of Major Organisations | 1112 | 393/82517 | 108/17799 | 1.17 | 0.99 | 1.38 | 1.66E-01 |
| Database Assistants/Clerks | 4136 | 125/82785 | 28/17879 | 1.16 | 0.83 | 1.64 | 5.44E-01 |
| Roundsmen/Women and Van Salespersons | 7123 | 29/82881 | 11/17896 | 1.16 | 0.73 | 1.83 | 6.85E-01 |
| Hairdressers, Barbers | 6221 | 241/82669 | 47/17860 | 1.15 | 0.89 | 1.50 | 4.51E-01 |
| Prison Service Officers (Below Principal Officer) | 3314 | 73/82837 | 26/17881 | 1.15 | 0.83 | 1.61 | 5.62E-01 |
| Motor Mechanics, Auto Engineers | 5231 | 208/82702 | 80/17827 | 1.15 | 0.96 | 1.38 | 2.60E-01 |
| Transport Operatives NEC | 8219 | 25/82885 | 10/17897 | 1.15 | 0.70 | 1.89 | 7.30E-01 |
| Police Officers (Sergeant and Below) | 3312 | 397/82513 | 128/17779 | 1.14 | 0.98 | 1.32 | 2.21E-01 |
| Kitchen and Catering Assistants | 9223 | 267/82643 | 58/17849 | 1.13 | 0.89 | 1.44 | 4.65E-01 |
| Planning and Quality Control Engineers | 2128 | 104/82806 | 34/17873 | 1.13 | 0.85 | 1.51 | 5.63E-01 |
| Sports Coaches, Instructors and Officials | 3442 | 93/82817 | 22/17885 | 1.12 | 0.78 | 1.62 | 6.86E-01 |
| Fire Service Officers (Leading Fire Officer and Below) | 3313 | 125/82785 | 49/17858 | 1.12 | 0.89 | 1.42 | 5.10E-01 |
| Civil Service Administrative Officers and Assistants | 4112 | 623/82287 | 144/17763 | 1.12 | 0.97 | 1.29 | 2.66E-01 |
| Retail Cashiers and Check-Out Operators | 7112 | 200/82710 | 36/17871 | 1.11 | 0.83 | 1.49 | 6.29E-01 |
| Market and Street Traders and Assistants | 7124 | 28/82882 | 7/17900 | 1.11 | 0.59 | 2.07 | 8.70E-01 |
| Cleaners, Domestics | 9233 | 483/82427 | 112/17795 | 1.11 | 0.94 | 1.30 | 3.81E-01 |
| Nursing Auxiliaries and Assistants | 6111 | 356/82554 | 68/17839 | 1.10 | 0.89 | 1.37 | 5.30E-01 |
| Gardeners and Groundsmen/Groundswomen | 5113 | 356/82554 | 110/17797 | 1.10 | 0.94 | 1.30 | 3.94E-01 |
| Careers Advisers and Vocational Guidance Specialists | 3564 | 102/82808 | 20/17887 | 1.10 | 0.75 | 1.62 | 7.82E-01 |
| Broadcasting Associate Professionals | 3432 | 206/82704 | 55/17852 | 1.10 | 0.86 | 1.39 | 6.01E-01 |
| Marketing and Sales Managers | 1132 | 1766/81144 | 486/17421 | 1.09 | 1.01 | 1.18 | 9.10E-02 |
| Property, Housing and Land Managers | 1231 | 568/82342 | 145/17762 | 1.09 | 0.94 | 1.26 | 3.94E-01 |
| Financial Managers and Chartered Secretaries | 1131 | 1034/81876 | 248/17659 | 1.09 | 0.97 | 1.22 | 2.66E-01 |
| Senior Officials In Local Government | 1113 | 222/82688 | 55/17852 | 1.09 | 0.86 | 1.37 | 6.35E-01 |
| Waiters, Waitresses | 9224 | 46/82864 | 10/17897 | 1.08 | 0.62 | 1.89 | 8.86E-01 |
| Merchandisers and Window Dressers | 7125 | 52/82858 | 10/17897 | 1.08 | 0.63 | 1.84 | 8.86E-01 |
| Vocational and Industrial Trainers and Instructors | 3563 | 453/82457 | 103/17804 | 1.08 | 0.90 | 1.29 | 5.73E-01 |
| School Crossing Patrol Attendants | 9243 | 20/82890 | 5/17902 | 1.07 | 0.52 | 2.22 | 9.32E-01 |
| Printing Machine Minders and Assistants | 9133 | 40/82870 | 13/17894 | 1.07 | 0.69 | 1.65 | 8.76E-01 |
| Civil Service Executive Officers | 4111 | 604/82306 | 139/17768 | 1.07 | 0.92 | 1.24 | 5.48E-01 |
| Security Guards and Related Occupations | 9241 | 251/82659 | 87/17820 | 1.06 | 0.89 | 1.27 | 6.66E-01 |
| Financial and Accounting Technicians | 3537 | 83/82827 | 18/17889 | 1.06 | 0.71 | 1.58 | 8.86E-01 |
| Undertakers and Mortuary Assistants | 6291 | 37/82873 | 10/17897 | 1.05 | 0.61 | 1.81 | 9.33E-01 |
| Actors, Entertainers | 3413 | 122/82788 | 34/17873 | 1.05 | 0.78 | 1.40 | 8.70E-01 |
| Local Government Clerical Officers and Assistants | 4113 | 667/82243 | 128/17779 | 1.05 | 0.90 | 1.22 | 7.14E-01 |
| Social Workers | 2442 | 579/82331 | 107/17800 | 1.04 | 0.88 | 1.23 | 7.92E-01 |
| Production, Works and Maintenance Managers | 1121 | 2002/80908 | 543/17364 | 1.04 | 0.96 | 1.12 | 4.83E-01 |
| Shelf Fillers | 9251 | 30/82880 | 10/17897 | 1.04 | 0.66 | 1.63 | 9.33E-01 |
| Protective Service Associate Professionals NEC | 3319 | 53/82857 | 13/17894 | 1.04 | 0.63 | 1.71 | 9.44E-01 |
| Housing and Welfare Officers | 3232 | 602/82308 | 112/17795 | 1.03 | 0.87 | 1.22 | 8.56E-01 |
| Furniture Makers, Other Craft Woodworkers | 5492 | 73/82837 | 22/17885 | 1.03 | 0.72 | 1.47 | 9.35E-01 |
| Legal Secretaries | 4212 | 172/82738 | 23/17884 | 1.02 | 0.70 | 1.50 | 9.48E-01 |
| Metal Machining Setters and Setter-Operators | 5221 | 72/82838 | 25/17882 | 1.02 | 0.73 | 1.43 | 9.47E-01 |
| Metal Working Machine Operatives | 8125 | 377/82533 | 117/17790 | 1.02 | 0.87 | 1.19 | 8.96E-01 |
| Plastics Process Operatives | 8116 | 23/82887 | 9/17898 | 1.02 | 0.59 | 1.75 | 9.67E-01 |
| Personal Assistants and Other Secretaries | 4215 | 1579/81331 | 184/17723 | 1.01 | 0.88 | 1.16 | 9.33E-01 |
| Conference and Exhibition Managers | 1222 | 47/82863 | 9/17898 | 1.01 | 0.57 | 1.79 | 9.81E-01 |
| Driving Instructors | 8215 | 149/82761 | 44/17863 | 1.01 | 0.77 | 1.32 | 9.62E-01 |
| Sales and Retail Assistants | 7111 | 1212/81698 | 206/17701 | 1.01 | 0.89 | 1.14 | 9.33E-01 |
| Hospital and Health Service Managers | 1181 | 443/82467 | 76/17831 | 1.01 | 0.82 | 1.24 | 9.61E-01 |
| Ship and Hovercraft Officers | 3513 | 29/82881 | 10/17897 | 1.01 | 0.57 | 1.78 | 9.84E-01 |
| Engineering Technicians | 3113 | 178/82732 | 55/17852 | 1.01 | 0.80 | 1.27 | 9.65E-01 |
| Officers of Non-Governmental Organisations | 4114 | 259/82651 | 47/17860 | 1.01 | 0.78 | 1.30 | 9.67E-01 |
| Medical and Dental Technicians | 3218 | 147/82763 | 30/17877 | 1.00 | 0.73 | 1.37 | 9.95E-01 |
| Car Park Attendants | 9245 | 14/82896 | 6/17901 | 1.00 | 0.49 | 2.01 | 9.95E-01 |
| Assemblers and Routine Operatives NEC | 8139 | 61/82849 | 19/17888 | 1.00 | 0.67 | 1.48 | 9.87E-01 |
| Senior Officials of Special Interest Organisations | 1114 | 325/82585 | 63/17844 | 0.99 | 0.80 | 1.24 | 9.79E-01 |
| Storage and Warehouse Managers | 1162 | 172/82738 | 53/17854 | 0.99 | 0.77 | 1.26 | 9.53E-01 |
| Electrical/Electronics Engineers NEC | 5249 | 317/82593 | 95/17812 | 0.99 | 0.83 | 1.18 | 9.41E-01 |
| Senior Officials In National Government | 1111 | 253/82657 | 51/17856 | 0.99 | 0.77 | 1.27 | 9.48E-01 |
| Social Services Managers | 1184 | 230/82680 | 43/17864 | 0.98 | 0.75 | 1.29 | 9.47E-01 |
| Houseparents and Residential Wardens | 6114 | 123/82787 | 21/17886 | 0.98 | 0.66 | 1.46 | 9.61E-01 |
| Personnel, Training and Industrial Relations Managers | 1135 | 648/82262 | 121/17786 | 0.98 | 0.83 | 1.15 | 9.08E-01 |
| Bus and Coach Drivers | 8213 | 222/82688 | 73/17834 | 0.97 | 0.80 | 1.18 | 8.86E-01 |
| Legal Associate Professionals | 3520 | 141/82769 | 25/17882 | 0.97 | 0.68 | 1.38 | 9.35E-01 |
| Finance and Investment Analysts/Advisers | 3534 | 495/82415 | 117/17790 | 0.97 | 0.82 | 1.14 | 8.54E-01 |
| Probation Officers | 2443 | 96/82814 | 21/17886 | 0.97 | 0.66 | 1.42 | 9.33E-01 |
| Personnel and Industrial Relations Officers | 3562 | 365/82545 | 57/17850 | 0.97 | 0.76 | 1.23 | 8.84E-01 |
| Youth and Community Workers | 3231 | 192/82718 | 38/17869 | 0.96 | 0.73 | 1.27 | 8.91E-01 |
| Inspectors of Factories, Utilities and Trading Standards | 3565 | 57/82853 | 12/17895 | 0.96 | 0.58 | 1.57 | 9.33E-01 |
| Ambulance Staff (Excluding Paramedics) | 6112 | 49/82861 | 12/17895 | 0.96 | 0.58 | 1.57 | 9.33E-01 |
| Customer Care Occupations | 7212 | 469/82441 | 87/17820 | 0.96 | 0.79 | 1.16 | 7.96E-01 |
| Quantity Surveyors | 2433 | 206/82704 | 51/17856 | 0.95 | 0.74 | 1.22 | 8.48E-01 |
| Business and Related Associate Professionals NEC | 3539 | 317/82593 | 59/17848 | 0.95 | 0.76 | 1.20 | 8.29E-01 |
| Travel Agency Managers | 1226 | 27/82883 | 5/17902 | 0.95 | 0.44 | 2.05 | 9.47E-01 |
| Transport and Distribution Clerks | 4134 | 145/82765 | 34/17873 | 0.95 | 0.71 | 1.27 | 8.54E-01 |
| Engineering Professionals NEC | 2129 | 319/82591 | 77/17830 | 0.93 | 0.77 | 1.13 | 6.32E-01 |
| Farm Workers | 9111 | 37/82873 | 7/17900 | 0.93 | 0.49 | 1.76 | 9.11E-01 |
| Originators, Compositors and Print Preparers | 5421 | 26/82884 | 6/17901 | 0.93 | 0.44 | 1.94 | 9.30E-01 |
| Purchasing Managers | 1133 | 105/82805 | 24/17883 | 0.93 | 0.65 | 1.33 | 8.17E-01 |
| Architectural Technologists and Town Planning Technicians | 3121 | 61/82849 | 14/17893 | 0.93 | 0.58 | 1.48 | 8.70E-01 |
| Solicitors and Lawyers, Judges and Coroners | 2411 | 899/82011 | 178/17729 | 0.92 | 0.80 | 1.05 | 3.81E-01 |
| Train Drivers | 3514 | 48/82862 | 15/17892 | 0.91 | 0.58 | 1.45 | 8.34E-01 |
| Fishing and Agriculture Related Occupations NEC | 9119 | 30/82880 | 8/17899 | 0.91 | 0.51 | 1.63 | 8.70E-01 |
| Authors, Writers | 3412 | 418/82492 | 72/17835 | 0.91 | 0.74 | 1.12 | 5.32E-01 |
| Recycling and Refuse Disposal Managers | 1235 | 27/82883 | 6/17901 | 0.90 | 0.44 | 1.84 | 8.81E-01 |
| Childminders and Related Occupations | 6122 | 193/82717 | 23/17884 | 0.89 | 0.61 | 1.32 | 7.17E-01 |
| Quality Assurance Managers | 1141 | 151/82759 | 30/17877 | 0.89 | 0.65 | 1.24 | 6.48E-01 |
| Filing and Other Records Assistants/Clerks | 4131 | 323/82587 | 49/17858 | 0.89 | 0.70 | 1.14 | 5.32E-01 |
| General Office Assistants/Clerks | 4150 | 2586/80324 | 338/17569 | 0.89 | 0.80 | 0.98 | 8.37E-02 |
| Air Transport Operatives | 8218 | 45/82865 | 11/17896 | 0.88 | 0.53 | 1.48 | 7.86E-01 |
| Launderers, Dry Cleaners, Pressers | 9234 | 34/82876 | 5/17902 | 0.88 | 0.39 | 1.98 | 8.76E-01 |
| Photographers and Audio-Visual Equipment Operators | 3434 | 213/82697 | 51/17856 | 0.88 | 0.69 | 1.11 | 4.46E-01 |
| Registrars and Senior Administrators of Educational Establishments | 2317 | 525/82385 | 69/17838 | 0.87 | 0.70 | 1.09 | 3.81E-01 |
| Plant and Machine Operatives NEC | 8129 | 28/82882 | 7/17900 | 0.87 | 0.46 | 1.63 | 8.04E-01 |
| Product, Clothing and Related Designers | 3422 | 235/82675 | 40/17867 | 0.87 | 0.65 | 1.17 | 5.26E-01 |
| Advertising and Public Relations Managers | 1134 | 131/82779 | 23/17884 | 0.87 | 0.59 | 1.28 | 6.29E-01 |
| Accounts and Wages Clerks, Book-Keepers, Other Financial Clerks | 4122 | 2209/80701 | 327/17580 | 0.87 | 0.78 | 0.96 | 2.93E-02 |
| Public Service Associate Professionals | 3561 | 739/82171 | 123/17784 | 0.87 | 0.74 | 1.02 | 1.96E-01 |
| Public Service Administrative Professionals | 2441 | 429/82481 | 78/17829 | 0.86 | 0.70 | 1.06 | 3.14E-01 |
| Education Officers, School Inspectors | 2313 | 230/82680 | 35/17872 | 0.86 | 0.63 | 1.17 | 5.03E-01 |
| Chartered Surveyors (Not Quantity Surveyors) | 2434 | 530/82380 | 116/17791 | 0.86 | 0.73 | 1.01 | 1.66E-01 |
| Insurance Underwriters | 3533 | 73/82837 | 13/17894 | 0.86 | 0.52 | 1.41 | 6.91E-01 |
| Occupational Hygienists and Safety Officers | 3567 | 226/82684 | 48/17859 | 0.86 | 0.66 | 1.11 | 3.94E-01 |
| Playgroup Leaders/Assistants | 6123 | 66/82844 | 7/17900 | 0.86 | 0.43 | 1.71 | 7.98E-01 |
| Credit Controllers | 4121 | 88/82822 | 13/17894 | 0.85 | 0.52 | 1.39 | 6.80E-01 |
| Computer Engineers, Installation and Maintenance | 5245 | 124/82786 | 28/17879 | 0.85 | 0.62 | 1.18 | 5.10E-01 |
| Dental Nurses | 6113 | 65/82845 | 7/17900 | 0.85 | 0.42 | 1.71 | 7.92E-01 |
| Management Consultants, Actuaries, Economists and Statisticians | 2423 | 986/81924 | 180/17727 | 0.85 | 0.74 | 0.97 | 6.42E-02 |
| Environmental Health Officers | 3568 | 77/82833 | 15/17892 | 0.85 | 0.54 | 1.34 | 6.33E-01 |
| Receptionists | 4216 | 755/82155 | 77/17830 | 0.85 | 0.68 | 1.05 | 2.60E-01 |
| Legal Professionals NEC | 2419 | 69/82841 | 10/17897 | 0.84 | 0.48 | 1.48 | 7.00E-01 |
| Taxation Experts | 3535 | 157/82753 | 26/17881 | 0.84 | 0.59 | 1.20 | 5.03E-01 |
| Travel Agents | 6212 | 97/82813 | 14/17893 | 0.84 | 0.51 | 1.37 | 6.33E-01 |
| Draughtspersons | 3122 | 195/82715 | 39/17868 | 0.84 | 0.63 | 1.11 | 3.81E-01 |
| Further Education Teaching Professionals | 2312 | 888/82022 | 138/17769 | 0.83 | 0.71 | 0.97 | 6.26E-02 |
| It User Support Technicians | 3132 | 91/82819 | 19/17888 | 0.83 | 0.55 | 1.25 | 5.32E-01 |
| Management Accountants | 2422 | 83/82827 | 15/17892 | 0.82 | 0.51 | 1.32 | 5.73E-01 |
| Electrical Engineers | 2123 | 195/82715 | 45/17862 | 0.82 | 0.63 | 1.07 | 2.75E-01 |
| Financial Institution Managers | 1151 | 560/82350 | 99/17808 | 0.82 | 0.68 | 0.98 | 1.02E-01 |
| Nurses | 3211 | 2121/80789 | 241/17666 | 0.82 | 0.72 | 0.92 | 5.99E-03 |
| Bakers, Flour Confectioners | 5432 | 42/82868 | 8/17899 | 0.81 | 0.44 | 1.51 | 6.60E-01 |
| Artists | 3411 | 163/82747 | 26/17881 | 0.81 | 0.57 | 1.16 | 4.06E-01 |
| Information and Communication Technology Managers | 1136 | 1224/81686 | 236/17671 | 0.81 | 0.72 | 0.91 | 3.12E-03 |
| Chartered and Certified Accountants | 2421 | 821/82089 | 139/17768 | 0.81 | 0.69 | 0.94 | 3.12E-02 |
| Educational Assistants | 6124 | 1075/81835 | 125/17782 | 0.81 | 0.69 | 0.95 | 4.40E-02 |
| Secondary Education Teaching Professionals | 2314 | 2678/80232 | 379/17528 | 0.80 | 0.73 | 0.88 | 5.92E-05 |
| Electronics Engineers | 2124 | 171/82739 | 35/17872 | 0.80 | 0.59 | 1.08 | 2.87E-01 |
| Archivists and Curators | 2452 | 82/82828 | 13/17894 | 0.80 | 0.47 | 1.35 | 5.58E-01 |
| Chemists | 2111 | 173/82737 | 32/17875 | 0.80 | 0.59 | 1.08 | 2.84E-01 |
| Bookbinders and Print Finishers | 5423 | 50/82860 | 11/17896 | 0.79 | 0.46 | 1.35 | 5.48E-01 |
| Teaching Professionals NEC | 2319 | 854/82056 | 108/17799 | 0.78 | 0.66 | 0.93 | 3.12E-02 |
| Counter Clerks | 4123 | 537/82373 | 63/17844 | 0.78 | 0.62 | 0.98 | 1.04E-01 |
| Therapists NEC | 3229 | 466/82444 | 51/17856 | 0.78 | 0.60 | 1.01 | 1.52E-01 |
| Medical Secretaries | 4211 | 404/82506 | 38/17869 | 0.77 | 0.57 | 1.05 | 2.12E-01 |
| Speech and Language Therapists | 3223 | 100/82810 | 10/17897 | 0.77 | 0.43 | 1.40 | 5.52E-01 |
| Pharmaceutical Dispensers | 3217 | 87/82823 | 9/17898 | 0.77 | 0.42 | 1.43 | 5.63E-01 |
| Estimators, Valuers and Assessors | 3531 | 180/82730 | 34/17873 | 0.76 | 0.56 | 1.04 | 1.96E-01 |
| It Strategy and Planning Professionals | 2131 | 802/82108 | 147/17760 | 0.76 | 0.66 | 0.89 | 3.07E-03 |
| Special Needs Education Teaching Professionals | 2316 | 446/82464 | 46/17861 | 0.75 | 0.58 | 0.99 | 1.21E-01 |
| It Operations Technicians | 3131 | 313/82597 | 56/17851 | 0.75 | 0.59 | 0.95 | 6.98E-02 |
| Mechanical Engineers | 2122 | 504/82406 | 94/17813 | 0.75 | 0.62 | 0.90 | 1.21E-02 |
| Housekeepers and Related Occupations | 6231 | 97/82813 | 12/17895 | 0.75 | 0.44 | 1.27 | 4.46E-01 |
| Healthcare Practice Managers | 1183 | 66/82844 | 7/17900 | 0.75 | 0.37 | 1.52 | 5.73E-01 |
| Fitness Instructors | 3443 | 107/82803 | 12/17895 | 0.73 | 0.43 | 1.23 | 3.94E-01 |
| Software Professionals | 2132 | 1041/81869 | 190/17717 | 0.72 | 0.63 | 0.82 | 1.91E-05 |
| Psychologists | 2212 | 184/82726 | 21/17886 | 0.72 | 0.48 | 1.08 | 2.42E-01 |
| Public Relations Officers | 3433 | 116/82794 | 15/17892 | 0.72 | 0.45 | 1.14 | 3.04E-01 |
| Call Centre Agents/Operators | 7211 | 90/82820 | 15/17892 | 0.72 | 0.45 | 1.13 | 2.94E-01 |
| Farmers | 5111 | 163/82747 | 26/17881 | 0.71 | 0.50 | 1.02 | 1.57E-01 |
| Graphic Designers | 3421 | 195/82715 | 30/17877 | 0.71 | 0.51 | 0.99 | 1.27E-01 |
| Pharmacists/Pharmacologists | 2213 | 223/82687 | 26/17881 | 0.71 | 0.49 | 1.01 | 1.55E-01 |
| Researchers NEC | 2329 | 221/82689 | 30/17877 | 0.70 | 0.50 | 0.98 | 1.21E-01 |
| Medical Radiographers | 3214 | 139/82771 | 13/17894 | 0.70 | 0.42 | 1.17 | 3.21E-01 |
| Quality Assurance Technicians | 3115 | 40/82870 | 6/17901 | 0.69 | 0.33 | 1.45 | 5.03E-01 |
| Building and Civil Engineering Technicians | 3114 | 79/82831 | 13/17894 | 0.68 | 0.41 | 1.12 | 2.66E-01 |
| Buyers and Purchasing Officers | 3541 | 177/82733 | 26/17881 | 0.68 | 0.48 | 0.97 | 1.04E-01 |
| Precision Instrument Makers and Repairers | 5224 | 78/82832 | 14/17893 | 0.67 | 0.42 | 1.06 | 2.02E-01 |
| Science and Engineering Technicians NEC | 3119 | 152/82758 | 22/17885 | 0.67 | 0.45 | 0.99 | 1.24E-01 |
| Typists | 4217 | 66/82844 | 5/17902 | 0.67 | 0.29 | 1.53 | 5.10E-01 |
| Market Research Interviewers | 4137 | 88/82822 | 10/17897 | 0.66 | 0.37 | 1.20 | 3.21E-01 |
| Goldsmiths, Silversmiths, Precious Stone Workers | 5495 | 35/82875 | 5/17902 | 0.65 | 0.29 | 1.44 | 4.51E-01 |
| Electrical/Electronics Technicians | 3112 | 57/82853 | 10/17897 | 0.65 | 0.37 | 1.13 | 2.66E-01 |
| Librarians | 2451 | 354/82556 | 35/17872 | 0.65 | 0.47 | 0.89 | 3.35E-02 |
| Research and Development Managers | 1137 | 237/82673 | 30/17877 | 0.64 | 0.46 | 0.90 | 4.21E-02 |
| Chiropodists | 3215 | 85/82825 | 8/17899 | 0.64 | 0.33 | 1.25 | 3.36E-01 |
| Horticultural Trades | 5112 | 34/82876 | 5/17902 | 0.63 | 0.28 | 1.42 | 4.34E-01 |
| Nursery Nurses | 6121 | 281/82629 | 22/17885 | 0.63 | 0.42 | 0.94 | 8.42E-02 |
| Dental Practitioners | 2215 | 181/82729 | 20/17887 | 0.62 | 0.41 | 0.94 | 8.68E-02 |
| Chemical Engineers | 2125 | 88/82822 | 13/17894 | 0.62 | 0.38 | 1.04 | 1.66E-01 |
| Civil Engineers | 2121 | 624/82286 | 92/17815 | 0.62 | 0.51 | 0.75 | 1.36E-05 |
| Design and Development Engineers | 2126 | 213/82697 | 31/17876 | 0.62 | 0.45 | 0.85 | 1.72E-02 |
| Higher Education Teaching Professionals | 2311 | 1856/81054 | 239/17668 | 0.62 | 0.55 | 0.70 | 1.88E-13 |
| Sewing Machinists | 8137 | 59/82851 | 5/17902 | 0.61 | 0.27 | 1.38 | 3.94E-01 |
| Laboratory Technicians | 3111 | 314/82596 | 34/17873 | 0.61 | 0.45 | 0.83 | 1.06E-02 |
| Midwives | 3212 | 213/82697 | 16/17891 | 0.61 | 0.38 | 0.98 | 1.21E-01 |
| Aircraft Pilots and Flight Engineers | 3512 | 45/82865 | 6/17901 | 0.61 | 0.28 | 1.31 | 3.61E-01 |
| Library Assistants/Clerks | 4135 | 240/82670 | 20/17887 | 0.60 | 0.40 | 0.91 | 6.23E-02 |
| Social Science Researchers | 2322 | 131/82779 | 13/17894 | 0.60 | 0.36 | 1.00 | 1.36E-01 |
| Scientific Researchers | 2321 | 138/82772 | 16/17891 | 0.59 | 0.38 | 0.93 | 7.97E-02 |
| Occupational Therapists | 3222 | 149/82761 | 12/17895 | 0.58 | 0.34 | 0.99 | 1.29E-01 |
| Natural Environment and Conservation Managers | 1212 | 54/82856 | 7/17900 | 0.57 | 0.29 | 1.13 | 2.35E-01 |
| Pensions and Insurance Clerks | 4132 | 212/82698 | 20/17887 | 0.54 | 0.36 | 0.81 | 1.60E-02 |
| Primary and Nursery Education Teaching Professionals | 2315 | 2572/80338 | 179/17728 | 0.53 | 0.46 | 0.61 | 2.94E-16 |
| Architects | 2431 | 435/82475 | 53/17854 | 0.52 | 0.40 | 0.67 | 6.99E-06 |
| Physiotherapists | 3221 | 300/82610 | 19/17888 | 0.52 | 0.33 | 0.80 | 1.67E-02 |
| Pharmacy Managers | 1182 | 56/82854 | 5/17902 | 0.51 | 0.23 | 1.15 | 2.30E-01 |
| Conservation and Environmental Protection Officers | 3551 | 72/82838 | 8/17899 | 0.51 | 0.27 | 0.98 | 1.25E-01 |
| School Midday Assistants | 9244 | 109/82801 | 7/17900 | 0.51 | 0.25 | 1.05 | 1.66E-01 |
| Town Planners | 2432 | 127/82783 | 14/17893 | 0.50 | 0.31 | 0.83 | 3.46E-02 |
| Biological Scientists and Biochemists | 2112 | 578/82332 | 49/17858 | 0.49 | 0.37 | 0.64 | 2.67E-06 |
| School Secretaries | 4213 | 335/82575 | 17/17890 | 0.45 | 0.28 | 0.71 | 4.43E-03 |
| Ophthalmic Opticians | 2214 | 94/82816 | 7/17900 | 0.40 | 0.20 | 0.84 | 5.89E-02 |
| Medical Practitioners | 2211 | 1074/81836 | 80/17827 | 0.40 | 0.32 | 0.50 | 2.48E-15 |
| Physicists, Geologists and Meteorologists | 2113 | 183/82727 | 15/17892 | 0.40 | 0.25 | 0.65 | 1.86E-03 |
| Clergy | 2444 | 374/82536 | 18/17889 | 0.20 | 0.13 | 0.32 | 2.36E-10 |
| ^NEC - Not Elsewhere Classified; NCO - non-commissioned officer^ |  |  |  |  |  |  |  |

**Table S2**: Trend for employment length in occupations associated with increased risk of heavy alcohol consumption

| Job | Job code | Trend direction | P_Trend_ |
| --- | --- | --- | --- |
| PUBLICANS AND MANAGERS OF LICENSED PREMISES | 1224 | Negative | 9.9E-04 |
| INDUSTRIAL CLEANING PROCESS OCCUPATIONS | 9132 | NS | 0.11 |
| PLASTERERS | 5321 | Positive | 3.1E-08 |
| SPORTS AND LEISURE ASSISTANTS | 6211 | NS | 0.70 |
| BAR STAFF | 9225 | Negative | 3.6E-05 |
| REFUSE AND SALVAGE OCCUPATIONS | 9235 | NS | 0.98 |
| WEIGHERS, GRADERS, SORTERS | 8134 | NS | 0.17 |
| AUTO ELECTRICIANS | 5233 | NS | 0.16 |
| ROOFERS, ROOF TILERS AND SLATERS | 5313 | Negative | 1.7E-06 |
| VEHICLE BODY BUILDERS AND REPAIRERS | 5232 | Positive | 6.6E-03 |
| GLAZIERS, WINDOW FABRICATORS AND FITTERS | 5316 | NS | 0.10 |
| PIPE FITTERS | 5216 | Positive | 7.0E-03 |
| BRICKLAYERS, MASONS | 5312 | Positive | 6.8E-16 |
| FLOORERS AND WALL TILERS | 5322 | Positive | 4.0E-13 |
| STEEL ERECTORS | 5311 | NS | 0.68 |
| BEAUTICIANS AND RELATED OCCUPATIONS | 6222 | NS | 0.99 |
| CONSTRUCTION TRADES NEC | 5319 | Positive | 1.4E-12 |
| ASSEMBLERS (VEHICLES AND METAL GOODS) | 8132 | Positive | 6.8E-03 |
| METAL PLATE WORKERS, SHIPWRIGHTS, RIVETERS | 5214 | NS | 0.68 |
| PLUMBERS, HEATING AND VENTILATING ENGINEERS | 5314 | Positive | 2.6E-21 |
| CHEMICAL AND RELATED PROCESS OPERATIVES | 8114 | Positive | 1.9E-05 |
| SCAFFOLDERS, STAGERS, RIGGERS | 8141 | NS | 0.26 |
| ROAD CONSTRUCTION OPERATIVES | 8142 | NS | 0.44 |
| CONSTRUCTION OPERATIVES NEC | 8149 | Positive | 1.0E-04 |
| FORK-LIFT TRUCK DRIVERS | 8222 | Negative | 3.4E-03 |
| LABOURERS IN BUILDING AND WOODWORKING TRADES | 9121 | Negative | 9.0E-11 |
| PAINTERS AND DECORATORS | 5323 | Positive | 1.5E-11 |
| WELDING TRADES | 5215 | NS | 0.64 |
| SHOPKEEPERS AND WHOLESALE/RETAIL DEALERS | 1234 | Positive | 4.8E-13 |
| PRINTERS | 5422 | Positive | 1.4E-03 |
| CARPENTERS AND JOINERS | 5315 | Positive | 5.2E-25 |
| FOOD, DRINK AND TOBACCO PROCESS OPERATIVES | 8111 | NS | 0.46 |
| WINDOW CLEANERS | 9231 | Positive | 3.7E-05 |
| RESTAURANT AND CATERING MANAGERS | 1223 | NS | 0.52 |
| CARETAKERS | 6232 | Negative | 4.0E-10 |
| OTHER GOODS HANDLING AND STORAGE OCCUPATIONS NEC | 9149 | Negative | 4.5E-13 |
| COMPANY SECRETARIES | 4214 | NS | 0.69 |
| CUSTOMER CARE MANAGERS | 1142 | NS | 0.32 |
| ELECTRICIANS, ELECTRICAL FITTERS | 5241 | Positive | 7.0E-08 |
| LABOURERS IN PROCESS AND PLANT OPERATIONS NEC | 9139 | NS | 0.62 |
| METAL WORKING PRODUCTION AND MAINTENANCE FITTERS | 5223 | Positive | 4.1E-08 |
| POSTAL WORKERS, MAIL SORTERS, MESSENGERS, COURIERS | 9211 | NS | 0.12 |
| BROKERS | 3532 | NS | 0.13 |
| TRANSPORT AND DISTRIBUTION MANAGERS | 1161 | NS | 0.05 |
| MANAGERS IN CONSTRUCTION | 1122 | NS | 0.06 |
| CHEFS, COOKS | 5434 | Negative | 3.1E-05 |
| HEAVY GOODS VEHICLE DRIVERS | 8211 | Negative | 6.9E-12 |
| VAN DRIVERS | 8212 | Negative | 7.6E-35 |
| CARE ASSISTANTS AND HOME CARERS | 6115 | Negative | 1.2E-36 |
| OFFICE MANAGERS | 1152 | Negative | 5.2E-03 |
| SALES REPRESENTATIVES | 3542 | Negative | 5.6E-08 |

Negative trend = negative linear trend for time employed and proportion of cases; Positive trend = positive linear trend for time employed and proportion of cases.

**Table S3:** Trend for employment length in occupations associated with decreased risk of heavy alcohol consumption

| Job | Job code | Trend direction | P_Trend_ |
| --- | --- | --- | --- |
| ACCOUNTS AND WAGES CLERKS, BOOK-KEEPERS, OTHER FINANCIAL CLERKS | 4122 | Negative | 6.1E-12 |
| NURSES | 3211 | NS | 0.19 |
| INFORMATION AND COMMUNICATION TECHNOLOGY MANAGERS | 1136 | NS | 0.72 |
| CHARTERED AND CERTIFIED ACCOUNTANTS | 2421 | NS | 0.46 |
| EDUCATIONAL ASSISTANTS | 6124 | Negative | 1.6E-49 |
| SECONDARY EDUCATION TEACHING PROFESSIONALS | 2314 | Positive | 1.3E-42 |
| TEACHING PROFESSIONALS NEC | 2319 | Negative | 4.8E-17 |
| IT STRATEGY AND PLANNING PROFESSIONALS | 2131 | Negative | 2.2E-05 |
| MECHANICAL ENGINEERS | 2122 | Positive | 7.2E-04 |
| SOFTWARE PROFESSIONALS | 2132 | NS | 0.78 |
| LIBRARIANS | 2451 | Positive | 0.03 |
| RESEARCH AND DEVELOPMENT MANAGERS | 1137 | NS | 0.51 |
| CIVIL ENGINEERS | 2121 | Positive | 9.5E-05 |
| DESIGN AND DEVELOPMENT ENGINEERS | 2126 | NS | 0.10 |
| HIGHER EDUCATION TEACHING PROFESSIONALS | 2311 | Positive | 2.8E-07 |
| LABORATORY TECHNICIANS | 3111 | NS | 0.24 |
| PENSIONS AND INSURANCE CLERKS | 4132 | NS | 0.05 |
| PRIMARY AND NURSERY EDUCATION TEACHING PROFESSIONALS | 2315 | Positive | 1.3E-16 |
| ARCHITECTS | 2431 | Positive | 1.4E-12 |
| PHYSIOTHERAPISTS | 3221 | Positive | 8.6E-03 |
| TOWN PLANNERS | 2432 | NS | 0.84 |
| BIOLOGICAL SCIENTISTS AND BIOCHEMISTS | 2112 | Positive | 5.1E-11 |
| SCHOOL SECRETARIES | 4213 | Negative | 2.5E-03 |
| MEDICAL PRACTITIONERS | 2211 | Positive | 2.8E-17 |
| PHYSICISTS, GEOLOGISTS AND METEOROLOGISTS | 2113 | Positive | 6.4E-06 |
| CLERGY | 2444 | NS | 0.90 |

Negative trend = negative linear trend for time employed and proportion of controls; Positive trend = positive linear trend for time employed and proportion of controls.

**Table S4: Occupations associated with alcohol consumption in males, ordered by PR**

| **Job** | **job code** | **Controls in job / not in job** | **Cases in job / not in job** | **PR** | **LCL** | **UCL** | **P-FDR** |
| --- | --- | --- | --- | --- | --- | --- | --- |
| Publicans and Managers of Licensed Premises | 1224 | 42/41747 | 98/12655 | 2.65 | 2.36 | 2.97 | 7.53E-59 |
| Industrial Cleaning Process Occupations | 9132 | 6/41783 | 9/12744 | 2.14 | 1.33 | 3.44 | 1.08E-02 |
| Auto Electricians | 5233 | 11/41778 | 10/12743 | 2.11 | 1.31 | 3.40 | 1.16E-02 |
| Bar Staff | 9225 | 12/41777 | 22/12731 | 2.10 | 1.60 | 2.75 | 1.63E-06 |
| Plasterers | 5321 | 31/41758 | 40/12713 | 2.09 | 1.68 | 2.61 | 1.87E-09 |
| Refuse and Salvage Occupations | 9235 | 11/41778 | 16/12737 | 2.03 | 1.45 | 2.83 | 3.00E-04 |
| Roofers, Roof Tilers and Slaters | 5313 | 25/41764 | 34/12719 | 1.89 | 1.54 | 2.32 | 1.76E-08 |
| Glaziers, Window Fabricators and Fitters | 5316 | 33/41756 | 33/12720 | 1.85 | 1.47 | 2.34 | 2.72E-06 |
| Vehicle Body Builders and Repairers | 5232 | 22/41767 | 20/12733 | 1.84 | 1.32 | 2.57 | 2.04E-03 |
| Pipe Fitters | 5216 | 23/41766 | 27/12726 | 1.79 | 1.37 | 2.34 | 1.67E-04 |
| Floorers and Wall Tilers | 5322 | 44/41745 | 40/12713 | 1.79 | 1.44 | 2.22 | 2.31E-06 |
| Bricklayers, Masons | 5312 | 82/41707 | 76/12677 | 1.78 | 1.51 | 2.11 | 8.24E-10 |
| Steel Erectors | 5311 | 19/41770 | 18/12735 | 1.78 | 1.29 | 2.46 | 3.14E-03 |
| Construction Trades NEC | 5319 | 381/41408 | 251/12502 | 1.64 | 1.49 | 1.80 | 9.70E-23 |
| Assemblers (Vehicles and Metal Goods) | 8132 | 39/41750 | 32/12721 | 1.63 | 1.26 | 2.10 | 1.34E-03 |
| Plumbers, Heating and Ventilating Engineers | 5314 | 335/41454 | 225/12528 | 1.61 | 1.46 | 1.78 | 1.10E-18 |
| Chemical and Related Process Operatives | 8114 | 63/41726 | 50/12703 | 1.60 | 1.31 | 1.96 | 4.73E-05 |
| Scaffolders, Stagers, Riggers | 8141 | 24/41765 | 23/12730 | 1.57 | 1.19 | 2.06 | 8.69E-03 |
| Construction Operatives NEC | 8149 | 96/41693 | 65/12688 | 1.56 | 1.29 | 1.88 | 3.14E-05 |
| Road Construction Operatives | 8142 | 31/41758 | 24/12729 | 1.55 | 1.17 | 2.05 | 1.16E-02 |
| Fork-Lift Truck Drivers | 8222 | 68/41721 | 54/12699 | 1.54 | 1.28 | 1.86 | 6.69E-05 |
| Labourers In Building and Woodworking Trades | 9121 | 123/41666 | 81/12672 | 1.53 | 1.29 | 1.81 | 9.41E-06 |
| Painters and Decorators | 5323 | 183/41606 | 111/12642 | 1.50 | 1.30 | 1.73 | 4.32E-07 |
| Food, Drink and Tobacco Process Operatives | 8111 | 51/41738 | 37/12716 | 1.48 | 1.15 | 1.91 | 1.16E-02 |
| Window Cleaners | 9231 | 33/41756 | 25/12728 | 1.48 | 1.10 | 1.99 | 4.24E-02 |
| Welding Trades | 5215 | 88/41701 | 60/12693 | 1.47 | 1.23 | 1.77 | 3.26E-04 |
| Carpenters and Joiners | 5315 | 387/41402 | 224/12529 | 1.46 | 1.32 | 1.62 | 3.93E-11 |
| Printers | 5422 | 55/41734 | 35/12718 | 1.46 | 1.14 | 1.87 | 1.62E-02 |
| Shopkeepers and Wholesale/Retail Dealers | 1234 | 227/41562 | 107/12646 | 1.45 | 1.24 | 1.69 | 3.14E-05 |
| Caretakers | 6232 | 135/41654 | 86/12667 | 1.39 | 1.19 | 1.63 | 3.71E-04 |
| Labourers In Process and Plant Operations NEC | 9139 | 153/41636 | 94/12659 | 1.37 | 1.17 | 1.61 | 6.44E-04 |
| Other Goods Handling and Storage Occupations NEC | 9149 | 284/41505 | 182/12571 | 1.36 | 1.22 | 1.52 | 1.29E-06 |
| Electricians, Electrical Fitters | 5241 | 648/41141 | 305/12448 | 1.34 | 1.23 | 1.47 | 5.60E-09 |
| Postal Workers, Mail Sorters, Messengers, Couriers | 9211 | 245/41544 | 136/12617 | 1.31 | 1.15 | 1.50 | 4.09E-04 |
| Metal Working Production and Maintenance Fitters | 5223 | 588/41201 | 282/12471 | 1.31 | 1.19 | 1.44 | 4.50E-07 |
| Managers In Construction | 1122 | 575/41214 | 239/12514 | 1.30 | 1.17 | 1.45 | 1.59E-05 |
| Transport and Distribution Managers | 1161 | 187/41602 | 81/12672 | 1.27 | 1.06 | 1.53 | 4.86E-02 |
| Heavy Goods Vehicle Drivers | 8211 | 415/41374 | 211/12542 | 1.25 | 1.12 | 1.40 | 4.88E-04 |
| Van Drivers | 8212 | 334/41455 | 150/12603 | 1.21 | 1.06 | 1.39 | 2.43E-02 |
| Management Consultants, Actuaries, Economists and Statisticians | 2423 | 695/41094 | 139/12614 | 0.80 | 0.68 | 0.93 | 1.70E-02 |
| Chartered and Certified Accountants | 2421 | 589/41200 | 114/12639 | 0.79 | 0.67 | 0.94 | 3.20E-02 |
| Information and Communication Technology Managers | 1136 | 976/40813 | 201/12552 | 0.79 | 0.70 | 0.90 | 1.67E-03 |
| Secondary Education Teaching Professionals | 2314 | 1076/40713 | 228/12525 | 0.78 | 0.69 | 0.88 | 3.71E-04 |
| Accounts and Wages Clerks, Book-Keepers, Other Financial Clerks | 4122 | 708/41081 | 148/12605 | 0.76 | 0.66 | 0.88 | 1.60E-03 |
| Mechanical Engineers | 2122 | 492/41297 | 93/12660 | 0.75 | 0.62 | 0.90 | 1.09E-02 |
| Financial Institution Managers | 1151 | 401/41388 | 70/12683 | 0.71 | 0.57 | 0.88 | 1.21E-02 |
| Public Service Administrative Professionals | 2441 | 271/41518 | 49/12704 | 0.71 | 0.55 | 0.92 | 4.56E-02 |
| Software Professionals | 2132 | 835/40954 | 163/12590 | 0.70 | 0.61 | 0.81 | 1.59E-05 |
| It Strategy and Planning Professionals | 2131 | 691/41098 | 120/12633 | 0.69 | 0.59 | 0.82 | 1.35E-04 |
| Teaching Professionals NEC | 2319 | 281/41508 | 48/12705 | 0.67 | 0.52 | 0.87 | 1.33E-02 |
| Design and Development Engineers | 2126 | 208/41581 | 31/12722 | 0.62 | 0.45 | 0.86 | 2.06E-02 |
| Civil Engineers | 2121 | 609/41180 | 89/12664 | 0.60 | 0.49 | 0.73 | 4.49E-06 |
| Research and Development Managers | 1137 | 163/41626 | 21/12732 | 0.56 | 0.38 | 0.83 | 2.16E-02 |
| Higher Education Teaching Professionals | 2311 | 1127/40662 | 162/12591 | 0.55 | 0.47 | 0.63 | 2.28E-14 |
| Architects | 2431 | 380/41409 | 47/12706 | 0.50 | 0.38 | 0.65 | 6.80E-06 |
| Primary and Nursery Education Teaching Professionals | 2315 | 345/41444 | 43/12710 | 0.49 | 0.37 | 0.65 | 9.41E-06 |
| Biological Scientists and Biochemists | 2112 | 264/41525 | 30/12723 | 0.47 | 0.34 | 0.67 | 1.67E-04 |
| Physicists, Geologists and Meteorologists | 2113 | 156/41633 | 14/12739 | 0.41 | 0.25 | 0.68 | 3.21E-03 |
| Town Planners | 2432 | 102/41687 | 9/12744 | 0.37 | 0.20 | 0.69 | 1.14E-02 |
| Medical Practitioners | 2211 | 581/41208 | 44/12709 | 0.32 | 0.24 | 0.42 | 1.46E-13 |
| Clergy | 2444 | 302/41487 | 15/12738 | 0.18 | 0.11 | 0.30 | 9.56E-10 |
| ^NEC - Not Elsewhere Classified^ |  |  |  |  |  |  |  |

**Table S5: Occupations associated with alcohol consumption in females, ordered by PR**

| **Job** | **job code** | **Controls in job / not in job** | **Cases in job / not in job** | **PR** | **LCL** | **UCL** | **P-FDR** |
| --- | --- | --- | --- | --- | --- | --- | --- |
| Publicans and Managers of Licensed Premises | 1224 | 26/41095 | 25/5129 | 3.79 | 2.82 | 5.09 | 1.91E-16 |
| Sports and Leisure Assistants | 6211 | 22/41099 | 10/5144 | 2.63 | 1.59 | 4.35 | 4.63E-03 |
| Storage and Warehouse Managers | 1162 | 18/41103 | 8/5146 | 2.48 | 1.41 | 4.37 | 1.77E-02 |
| Construction Trades NEC | 5319 | 27/41094 | 10/5144 | 2.26 | 1.30 | 3.93 | 3.07E-02 |
| Estate Agents, Auctioneers | 3544 | 47/41074 | 13/5141 | 2.24 | 1.38 | 3.63 | 1.69E-02 |
| Driving Instructors | 8215 | 28/41093 | 10/5144 | 2.22 | 1.35 | 3.64 | 1.77E-02 |
| Bar Staff | 9225 | 36/41085 | 14/5140 | 2.07 | 1.35 | 3.19 | 1.69E-02 |
| Restaurant and Catering Managers | 1223 | 98/41023 | 31/5123 | 1.95 | 1.44 | 2.64 | 9.49E-04 |
| Other Goods Handling and Storage Occupations NEC | 9149 | 44/41077 | 15/5139 | 1.89 | 1.23 | 2.90 | 3.07E-02 |
| Directors and Chief Executives of Major Organisations | 1112 | 65/41056 | 17/5137 | 1.83 | 1.19 | 2.81 | 3.99E-02 |
| Police Officers (Sergeant and Below) | 3312 | 61/41060 | 19/5135 | 1.77 | 1.20 | 2.63 | 3.19E-02 |
| It Strategy and Planning Professionals | 2131 | 111/41010 | 27/5127 | 1.70 | 1.21 | 2.39 | 2.25E-02 |
| Sales Representatives | 3542 | 202/40919 | 49/5105 | 1.67 | 1.30 | 2.13 | 2.15E-03 |
| Marketing Associate Professionals | 3543 | 142/40979 | 35/5119 | 1.65 | 1.22 | 2.24 | 1.69E-02 |
| Vocational and Industrial Trainers and Instructors | 3563 | 233/40888 | 52/5102 | 1.61 | 1.26 | 2.06 | 4.63E-03 |
| Retail and Wholesale Managers | 1163 | 187/40934 | 41/5113 | 1.58 | 1.20 | 2.09 | 1.69E-02 |
| Production, Works and Maintenance Managers | 1121 | 282/40839 | 56/5098 | 1.41 | 1.11 | 1.79 | 3.81E-02 |
| Care Assistants and Home Carers | 6115 | 610/40511 | 114/5040 | 1.36 | 1.15 | 1.60 | 9.30E-03 |
| Marketing and Sales Managers | 1132 | 430/40691 | 83/5071 | 1.34 | 1.10 | 1.64 | 3.07E-02 |
| Office Managers | 1152 | 633/40488 | 111/5043 | 1.32 | 1.11 | 1.56 | 1.77E-02 |
| Nurses | 3211 | 1962/39159 | 199/4955 | 0.82 | 0.72 | 0.94 | 3.07E-02 |
| Educational Assistants | 6124 | 996/40125 | 101/5053 | 0.77 | 0.64 | 0.93 | 4.41E-02 |
| Medical Practitioners | 2211 | 493/40628 | 36/5118 | 0.57 | 0.42 | 0.79 | 1.37E-02 |
| Primary and Nursery Education Teaching Professionals | 2315 | 2227/38894 | 136/5018 | 0.56 | 0.48 | 0.66 | 6.97E-10 |
| Physiotherapists | 3221 | 280/40841 | 17/5137 | 0.53 | 0.33 | 0.84 | 4.50E-02 |
| Biological Scientists and Biochemists | 2112 | 314/40807 | 19/5135 | 0.51 | 0.33 | 0.79 | 2.31E-02 |
| School Secretaries | 4213 | 321/40800 | 16/5138 | 0.46 | 0.29 | 0.74 | 1.69E-02 |
| ^NEC - Not Elsewhere Classified^ |  |  |  |  |  |  |  |

**Table S6: Sensitivity analysis outcomes for all occupations, ordered by PR**

| **Job** | **Job code** | **Controls in job / not in job** | **Cases in job / not in job** | **PR** | **LCL** | **UCL** | **P-FDR** |
| --- | --- | --- | --- | --- | --- | --- | --- |
| PUBLICANS AND MANAGERS OF LICENSED PREMISES | 1224 | 68/82842 | 123/17784 | 2.81 | 2.52 | 3.14 | 2.45E-73 |
| BRICKLAYERS, MASONS | 5312 | 82/82828 | 76/17831 | 1.80 | 1.52 | 2.14 | 1.11E-10 |
| CONSTRUCTION TRADES NEC | 5319 | 408/82502 | 261/17646 | 1.66 | 1.51 | 1.82 | 7.62E-25 |
| PLUMBERS, HEATING AND VENTILATING ENGINEERS | 5314 | 338/82572 | 226/17681 | 1.62 | 1.46 | 1.79 | 1.19E-19 |
| CHEMICAL AND RELATED PROCESS OPERATIVES | 8114 | 68/82842 | 50/17857 | 1.59 | 1.30 | 1.94 | 2.91E-05 |
| CONSTRUCTION OPERATIVES NEC | 8149 | 99/82811 | 65/17842 | 1.56 | 1.30 | 1.88 | 1.25E-05 |
| FORK-LIFT TRUCK DRIVERS | 8222 | 70/82840 | 54/17853 | 1.56 | 1.29 | 1.88 | 2.23E-05 |
| LABOURERS IN BUILDING AND WOODWORKING TRADES | 9121 | 124/82786 | 82/17825 | 1.56 | 1.32 | 1.84 | 1.59E-06 |
| PAINTERS AND DECORATORS | 5323 | 198/82712 | 114/17793 | 1.52 | 1.32 | 1.75 | 5.52E-08 |
| WELDING TRADES | 5215 | 88/82822 | 60/17847 | 1.50 | 1.24 | 1.80 | 7.71E-05 |
| SHOPKEEPERS AND WHOLESALE/RETAIL DEALERS | 1234 | 357/82553 | 132/17775 | 1.48 | 1.29 | 1.71 | 4.18E-07 |
| CARPENTERS AND JOINERS | 5315 | 388/82522 | 224/17683 | 1.47 | 1.32 | 1.63 | 5.78E-12 |
| RESTAURANT AND CATERING MANAGERS | 1223 | 195/82715 | 74/17833 | 1.43 | 1.18 | 1.73 | 1.09E-03 |
| CARETAKERS | 6232 | 156/82754 | 90/17817 | 1.42 | 1.21 | 1.66 | 5.60E-05 |
| OTHER GOODS HANDLING AND STORAGE OCCUPATIONS NEC | 9149 | 328/82582 | 197/17710 | 1.40 | 1.26 | 1.57 | 7.68E-09 |
| CUSTOMER CARE MANAGERS | 1142 | 173/82737 | 53/17854 | 1.37 | 1.09 | 1.73 | 1.81E-02 |
| ELECTRICIANS, ELECTRICAL FITTERS | 5241 | 650/82260 | 305/17602 | 1.36 | 1.24 | 1.49 | 5.12E-10 |
| LABOURERS IN PROCESS AND PLANT OPERATIONS NEC | 9139 | 196/82714 | 99/17808 | 1.34 | 1.15 | 1.57 | 8.85E-04 |
| METAL WORKING PRODUCTION AND MAINTENANCE FITTERS | 5223 | 596/82314 | 283/17624 | 1.32 | 1.20 | 1.46 | 7.40E-08 |
| POSTAL WORKERS, MAIL SORTERS, MESSENGERS, COURIERS | 9211 | 303/82607 | 147/17760 | 1.32 | 1.16 | 1.50 | 9.03E-05 |
| BROKERS | 3532 | 168/82742 | 63/17844 | 1.31 | 1.06 | 1.62 | 2.80E-02 |
| TRANSPORT AND DISTRIBUTION MANAGERS | 1161 | 214/82696 | 88/17819 | 1.31 | 1.10 | 1.56 | 8.58E-03 |
| MARKETING ASSOCIATE PROFESSIONALS | 3543 | 265/82645 | 67/17840 | 1.28 | 1.03 | 1.59 | 5.47E-02 |
| MANAGERS IN CONSTRUCTION | 1122 | 617/82293 | 241/17666 | 1.28 | 1.15 | 1.42 | 3.49E-05 |
| CHEFS, COOKS | 5434 | 314/82596 | 99/17808 | 1.27 | 1.07 | 1.50 | 1.49E-02 |
| HEAVY GOODS VEHICLE DRIVERS | 8211 | 423/82487 | 213/17694 | 1.27 | 1.14 | 1.41 | 8.73E-05 |
| VAN DRIVERS | 8212 | 351/82559 | 156/17751 | 1.25 | 1.10 | 1.43 | 2.84E-03 |
| CARE ASSISTANTS AND HOME CARERS | 6115 | 725/82185 | 169/17738 | 1.24 | 1.09 | 1.42 | 3.47E-03 |
| JOURNALISTS, NEWSPAPER AND PERIODICAL EDITORS | 3431 | 283/82627 | 78/17829 | 1.24 | 1.02 | 1.51 | 5.92E-02 |
| OFFICE MANAGERS | 1152 | 879/82031 | 194/17713 | 1.24 | 1.09 | 1.41 | 2.84E-03 |
| TELECOMMUNICATIONS ENGINEERS | 5242 | 176/82734 | 67/17840 | 1.23 | 1.01 | 1.50 | 6.69E-02 |
| STOCK CONTROL CLERKS | 4133 | 161/82749 | 53/17854 | 1.20 | 0.95 | 1.50 | 2.03E-01 |
| RETAIL AND WHOLESALE MANAGERS | 1163 | 538/82372 | 158/17749 | 1.18 | 1.03 | 1.36 | 3.49E-02 |
| MANAGERS AND PROPRIETORS IN OTHER SERVICES NEC | 1239 | 531/82379 | 144/17763 | 1.18 | 1.03 | 1.36 | 4.11E-02 |
| SALES REPRESENTATIVES | 3542 | 644/82266 | 189/17718 | 1.18 | 1.04 | 1.34 | 2.06E-02 |
| TAXI, CAB DRIVERS AND CHAUFFEURS | 8214 | 255/82655 | 104/17803 | 1.17 | 1.00 | 1.38 | 1.02E-01 |
| DIRECTORS AND CHIEF EXECUTIVES OF MAJOR ORGANISATIONS | 1112 | 393/82517 | 108/17799 | 1.17 | 0.99 | 1.38 | 1.17E-01 |
| MOTOR MECHANICS, AUTO ENGINEERS | 5231 | 208/82702 | 80/17827 | 1.15 | 0.96 | 1.38 | 2.00E-01 |
| POLICE OFFICERS (SERGEANT AND BELOW) | 3312 | 397/82513 | 128/17779 | 1.14 | 0.98 | 1.32 | 1.68E-01 |
| KITCHEN AND CATERING ASSISTANTS | 9223 | 267/82643 | 58/17849 | 1.13 | 0.89 | 1.44 | 4.11E-01 |
| CIVIL SERVICE ADMINISTRATIVE OFFICERS AND ASSISTANTS | 4112 | 623/82287 | 144/17763 | 1.12 | 0.97 | 1.29 | 2.03E-01 |
| CLEANERS, DOMESTICS | 9233 | 483/82427 | 112/17795 | 1.11 | 0.94 | 1.30 | 3.22E-01 |
| NURSING AUXILIARIES AND ASSISTANTS | 6111 | 356/82554 | 68/17839 | 1.10 | 0.89 | 1.37 | 4.82E-01 |
| GARDENERS AND GROUNDSMEN/GROUNDSWOMEN | 5113 | 356/82554 | 110/17797 | 1.10 | 0.94 | 1.30 | 3.33E-01 |
| BROADCASTING ASSOCIATE PROFESSIONALS | 3432 | 206/82704 | 55/17852 | 1.10 | 0.86 | 1.39 | 5.68E-01 |
| MARKETING AND SALES MANAGERS | 1132 | 1766/81144 | 486/17421 | 1.09 | 1.01 | 1.18 | 5.47E-02 |
| PROPERTY, HOUSING AND LAND MANAGERS | 1231 | 568/82342 | 145/17762 | 1.09 | 0.94 | 1.26 | 3.33E-01 |
| FINANCIAL MANAGERS AND CHARTERED SECRETARIES | 1131 | 1034/81876 | 248/17659 | 1.09 | 0.97 | 1.22 | 2.04E-01 |
| SENIOR OFFICIALS IN LOCAL GOVERNMENT | 1113 | 222/82688 | 55/17852 | 1.09 | 0.86 | 1.37 | 6.01E-01 |
| VOCATIONAL AND INDUSTRIAL TRAINERS AND INSTRUCTORS | 3563 | 453/82457 | 103/17804 | 1.08 | 0.90 | 1.29 | 5.46E-01 |
| CIVIL SERVICE EXECUTIVE OFFICERS | 4111 | 604/82306 | 139/17768 | 1.07 | 0.92 | 1.24 | 5.06E-01 |
| SECURITY GUARDS AND RELATED OCCUPATIONS | 9241 | 251/82659 | 87/17820 | 1.06 | 0.89 | 1.27 | 6.33E-01 |
| LOCAL GOVERNMENT CLERICAL OFFICERS AND ASSISTANTS | 4113 | 667/82243 | 128/17779 | 1.05 | 0.90 | 1.22 | 6.87E-01 |
| SOCIAL WORKERS | 2442 | 579/82331 | 107/17800 | 1.04 | 0.88 | 1.23 | 7.76E-01 |
| PRODUCTION, WORKS AND MAINTENANCE MANAGERS | 1121 | 2002/80908 | 543/17364 | 1.04 | 0.96 | 1.12 | 4.22E-01 |
| HOUSING AND WELFARE OFFICERS | 3232 | 602/82308 | 112/17795 | 1.03 | 0.87 | 1.22 | 8.25E-01 |
| METAL WORKING MACHINE OPERATIVES | 8125 | 377/82533 | 117/17790 | 1.02 | 0.87 | 1.19 | 8.79E-01 |
| PERSONAL ASSISTANTS AND OTHER SECRETARIES | 4215 | 1579/81331 | 184/17723 | 1.01 | 0.88 | 1.16 | 9.21E-01 |
| SALES AND RETAIL ASSISTANTS | 7111 | 1212/81698 | 206/17701 | 1.01 | 0.89 | 1.14 | 9.21E-01 |
| HOSPITAL AND HEALTH SERVICE MANAGERS | 1181 | 443/82467 | 76/17831 | 1.01 | 0.82 | 1.24 | 9.45E-01 |
| ENGINEERING TECHNICIANS | 3113 | 178/82732 | 55/17852 | 1.01 | 0.80 | 1.27 | 9.50E-01 |
| SENIOR OFFICIALS OF SPECIAL INTEREST ORGANISATIONS | 1114 | 325/82585 | 63/17844 | 0.99 | 0.80 | 1.24 | 9.64E-01 |
| STORAGE AND WAREHOUSE MANAGERS | 1162 | 172/82738 | 53/17854 | 0.99 | 0.77 | 1.26 | 9.44E-01 |
| ELECTRICAL/ELECTRONICS ENGINEERS NEC | 5249 | 317/82593 | 95/17812 | 0.99 | 0.83 | 1.18 | 9.29E-01 |
| SENIOR OFFICIALS IN NATIONAL GOVERNMENT | 1111 | 253/82657 | 51/17856 | 0.99 | 0.77 | 1.27 | 9.44E-01 |
| PERSONNEL, TRAINING AND INDUSTRIAL RELATIONS MANAGERS | 1135 | 648/82262 | 121/17786 | 0.98 | 0.83 | 1.15 | 8.85E-01 |
| BUS AND COACH DRIVERS | 8213 | 222/82688 | 73/17834 | 0.97 | 0.80 | 1.18 | 8.67E-01 |
| FINANCE AND INVESTMENT ANALYSTS/ADVISERS | 3534 | 495/82415 | 117/17790 | 0.97 | 0.82 | 1.14 | 8.25E-01 |
| PERSONNEL AND INDUSTRIAL RELATIONS OFFICERS | 3562 | 365/82545 | 57/17850 | 0.97 | 0.76 | 1.23 | 8.67E-01 |
| CUSTOMER CARE OCCUPATIONS | 7212 | 469/82441 | 87/17820 | 0.96 | 0.79 | 1.16 | 7.76E-01 |
| QUANTITY SURVEYORS | 2433 | 206/82704 | 51/17856 | 0.95 | 0.74 | 1.22 | 8.25E-01 |
| BUSINESS AND RELATED ASSOCIATE PROFESSIONALS NEC | 3539 | 317/82593 | 59/17848 | 0.95 | 0.76 | 1.20 | 8.11E-01 |
| ENGINEERING PROFESSIONALS NEC | 2129 | 319/82591 | 77/17830 | 0.93 | 0.77 | 1.13 | 5.98E-01 |
| SOLICITORS AND LAWYERS, JUDGES AND CORONERS | 2411 | 899/82011 | 178/17729 | 0.92 | 0.80 | 1.05 | 3.22E-01 |
| AUTHORS, WRITERS | 3412 | 418/82492 | 72/17835 | 0.91 | 0.74 | 1.12 | 4.84E-01 |
| GENERAL OFFICE ASSISTANTS/CLERKS | 4150 | 2586/80324 | 338/17569 | 0.89 | 0.80 | 0.98 | 4.91E-02 |
| PHOTOGRAPHERS AND AUDIO-VISUAL EQUIPMENT OPERATORS | 3434 | 213/82697 | 51/17856 | 0.88 | 0.69 | 1.11 | 3.89E-01 |
| REGISTRARS AND SENIOR ADMINISTRATORS OF EDUCATIONAL ESTABLISHMENTS | 2317 | 525/82385 | 69/17838 | 0.87 | 0.70 | 1.09 | 3.22E-01 |
| ACCOUNTS AND WAGES CLERKS, BOOK-KEEPERS, OTHER FINANCIAL CLERKS | 4122 | 2209/80701 | 327/17580 | 0.87 | 0.78 | 0.96 | 1.61E-02 |
| PUBLIC SERVICE ASSOCIATE PROFESSIONALS | 3561 | 739/82171 | 123/17784 | 0.87 | 0.74 | 1.02 | 1.45E-01 |
| PUBLIC SERVICE ADMINISTRATIVE PROFESSIONALS | 2441 | 429/82481 | 78/17829 | 0.86 | 0.70 | 1.06 | 2.56E-01 |
| CHARTERED SURVEYORS (NOT QUANTITY SURVEYORS) | 2434 | 530/82380 | 116/17791 | 0.86 | 0.73 | 1.01 | 1.18E-01 |
| MANAGEMENT CONSULTANTS, ACTUARIES, ECONOMISTS AND STATISTICIANS | 2423 | 986/81924 | 180/17727 | 0.85 | 0.74 | 0.97 | 3.74E-02 |
| RECEPTIONISTS | 4216 | 755/82155 | 77/17830 | 0.85 | 0.68 | 1.05 | 2.00E-01 |
| FURTHER EDUCATION TEACHING PROFESSIONALS | 2312 | 888/82022 | 138/17769 | 0.83 | 0.71 | 0.97 | 3.64E-02 |
| FINANCIAL INSTITUTION MANAGERS | 1151 | 560/82350 | 99/17808 | 0.82 | 0.68 | 0.98 | 6.11E-02 |
| NURSES | 3211 | 2121/80789 | 241/17666 | 0.82 | 0.72 | 0.92 | 2.84E-03 |
| INFORMATION AND COMMUNICATION TECHNOLOGY MANAGERS | 1136 | 1224/81686 | 236/17671 | 0.81 | 0.72 | 0.91 | 1.46E-03 |
| CHARTERED AND CERTIFIED ACCOUNTANTS | 2421 | 821/82089 | 139/17768 | 0.81 | 0.69 | 0.94 | 1.68E-02 |
| EDUCATIONAL ASSISTANTS | 6124 | 1075/81835 | 125/17782 | 0.81 | 0.69 | 0.95 | 2.43E-02 |
| SECONDARY EDUCATION TEACHING PROFESSIONALS | 2314 | 2678/80232 | 379/17528 | 0.80 | 0.73 | 0.88 | 2.60E-05 |
| TEACHING PROFESSIONALS NEC | 2319 | 854/82056 | 108/17799 | 0.78 | 0.66 | 0.93 | 1.68E-02 |
| COUNTER CLERKS | 4123 | 537/82373 | 63/17844 | 0.78 | 0.62 | 0.98 | 6.23E-02 |
| THERAPISTS NEC | 3229 | 466/82444 | 51/17856 | 0.78 | 0.60 | 1.01 | 1.03E-01 |
| IT STRATEGY AND PLANNING PROFESSIONALS | 2131 | 802/82108 | 147/17760 | 0.76 | 0.66 | 0.89 | 1.45E-03 |
| IT OPERATIONS TECHNICIANS | 3131 | 313/82597 | 56/17851 | 0.75 | 0.59 | 0.95 | 4.08E-02 |
| MECHANICAL ENGINEERS | 2122 | 504/82406 | 94/17813 | 0.75 | 0.62 | 0.90 | 6.33E-03 |
| SOFTWARE PROFESSIONALS | 2132 | 1041/81869 | 190/17717 | 0.72 | 0.63 | 0.82 | 8.79E-06 |
| CIVIL ENGINEERS | 2121 | 624/82286 | 92/17815 | 0.62 | 0.51 | 0.75 | 6.37E-06 |
| HIGHER EDUCATION TEACHING PROFESSIONALS | 2311 | 1856/81054 | 239/17668 | 0.62 | 0.55 | 0.70 | 6.12E-14 |
| PRIMARY AND NURSERY EDUCATION TEACHING PROFESSIONALS | 2315 | 2572/80338 | 179/17728 | 0.53 | 0.46 | 0.61 | 9.58E-17 |
| ARCHITECTS | 2431 | 435/82475 | 53/17854 | 0.52 | 0.40 | 0.67 | 3.34E-06 |
| MEDICAL PRACTITIONERS | 2211 | 1074/81836 | 80/17827 | 0.40 | 0.32 | 0.50 | 8.08E-16 |
| ^NEC - Not Elsewhere Classified; NCO - non-commissioned officer^ |  |  |  |  |  |  |  |

**Table S7: Sensitivity analysis outcomes for males, ordered by PR**

| **Job** | **Job code** | **Controls in job / not in job** | **Controls in job / not in job** | **PR** | **LCL** | **UCL** | **P-FDR** |
| --- | --- | --- | --- | --- | --- | --- | --- |
| BRICKLAYERS, MASONS | 5312 | 82/41707 | 76/12677 | 1.78 | 1.51 | 2.11 | 2.90E-10 |
| CONSTRUCTION TRADES NEC | 5319 | 381/41408 | 251/12502 | 1.64 | 1.49 | 1.80 | 4.87E-23 |
| PLUMBERS, HEATING AND VENTILATING ENGINEERS | 5314 | 335/41454 | 225/12528 | 1.61 | 1.46 | 1.78 | 4.14E-19 |
| CHEMICAL AND RELATED PROCESS OPERATIVES | 8114 | 63/41726 | 50/12703 | 1.60 | 1.31 | 1.96 | 1.93E-05 |
| CONSTRUCTION OPERATIVES NEC | 8149 | 96/41693 | 65/12688 | 1.56 | 1.29 | 1.88 | 1.31E-05 |
| FORK-LIFT TRUCK DRIVERS | 8222 | 68/41721 | 54/12699 | 1.54 | 1.28 | 1.86 | 2.66E-05 |
| LABOURERS IN BUILDING AND WOODWORKING TRADES | 9121 | 123/41666 | 81/12672 | 1.53 | 1.29 | 1.81 | 4.51E-06 |
| PAINTERS AND DECORATORS | 5323 | 183/41606 | 111/12642 | 1.50 | 1.30 | 1.73 | 1.83E-07 |
| WELDING TRADES | 5215 | 88/41701 | 60/12693 | 1.47 | 1.23 | 1.77 | 1.38E-04 |
| CARPENTERS AND JOINERS | 5315 | 387/41402 | 224/12529 | 1.46 | 1.32 | 1.62 | 1.48E-11 |
| SHOPKEEPERS AND WHOLESALE/RETAIL DEALERS | 1234 | 227/41562 | 107/12646 | 1.45 | 1.24 | 1.69 | 1.31E-05 |
| CARETAKERS | 6232 | 135/41654 | 86/12667 | 1.39 | 1.19 | 1.63 | 1.51E-04 |
| LABOURERS IN PROCESS AND PLANT OPERATIONS NEC | 9139 | 153/41636 | 94/12659 | 1.37 | 1.17 | 1.61 | 2.49E-04 |
| OTHER GOODS HANDLING AND STORAGE OCCUPATIONS NEC | 9149 | 284/41505 | 182/12571 | 1.36 | 1.22 | 1.52 | 5.05E-07 |
| ELECTRICIANS, ELECTRICAL FITTERS | 5241 | 648/41141 | 305/12448 | 1.34 | 1.23 | 1.47 | 2.34E-09 |
| POSTAL WORKERS, MAIL SORTERS, MESSENGERS, COURIERS | 9211 | 245/41544 | 136/12617 | 1.31 | 1.15 | 1.50 | 1.63E-04 |
| METAL WORKING PRODUCTION AND MAINTENANCE FITTERS | 5223 | 588/41201 | 282/12471 | 1.31 | 1.19 | 1.44 | 1.83E-07 |
| BROKERS | 3532 | 133/41656 | 56/12697 | 1.31 | 1.05 | 1.63 | 3.03E-02 |
| MANAGERS IN CONSTRUCTION | 1122 | 575/41214 | 239/12514 | 1.30 | 1.17 | 1.45 | 7.08E-06 |
| TRANSPORT AND DISTRIBUTION MANAGERS | 1161 | 187/41602 | 81/12672 | 1.27 | 1.06 | 1.53 | 2.33E-02 |
| CHEFS, COOKS | 5434 | 108/41681 | 60/12693 | 1.27 | 1.03 | 1.56 | 4.34E-02 |
| HEAVY GOODS VEHICLE DRIVERS | 8211 | 415/41374 | 211/12542 | 1.25 | 1.12 | 1.40 | 1.92E-04 |
| TELECOMMUNICATIONS ENGINEERS | 5242 | 173/41616 | 66/12687 | 1.23 | 1.01 | 1.50 | 7.78E-02 |
| JOURNALISTS, NEWSPAPER AND PERIODICAL EDITORS | 3431 | 132/41657 | 51/12702 | 1.23 | 0.97 | 1.55 | 1.46E-01 |
| MANAGERS AND PROPRIETORS IN OTHER SERVICES NEC | 1239 | 301/41488 | 109/12644 | 1.22 | 1.04 | 1.43 | 3.03E-02 |
| VAN DRIVERS | 8212 | 334/41455 | 150/12603 | 1.21 | 1.06 | 1.39 | 1.16E-02 |
| CARE ASSISTANTS AND HOME CARERS | 6115 | 115/41674 | 55/12698 | 1.16 | 0.95 | 1.42 | 2.25E-01 |
| MOTOR MECHANICS, AUTO ENGINEERS | 5231 | 208/41581 | 80/12673 | 1.15 | 0.96 | 1.37 | 2.07E-01 |
| TAXI, CAB DRIVERS AND CHAUFFEURS | 8214 | 246/41543 | 102/12651 | 1.14 | 0.97 | 1.35 | 1.81E-01 |
| OFFICE MANAGERS | 1152 | 246/41543 | 83/12670 | 1.14 | 0.95 | 1.37 | 2.45E-01 |
| SOCIAL WORKERS | 2442 | 129/41660 | 50/12703 | 1.13 | 0.90 | 1.42 | 4.00E-01 |
| FINANCIAL MANAGERS AND CHARTERED SECRETARIES | 1131 | 634/41155 | 198/12555 | 1.12 | 0.99 | 1.27 | 1.09E-01 |
| DIRECTORS AND CHIEF EXECUTIVES OF MAJOR ORGANISATIONS | 1112 | 328/41461 | 91/12662 | 1.10 | 0.92 | 1.32 | 4.00E-01 |
| RETAIL AND WHOLESALE MANAGERS | 1163 | 351/41438 | 117/12636 | 1.10 | 0.94 | 1.28 | 3.31E-01 |
| SALES AND RETAIL ASSISTANTS | 7111 | 234/41555 | 88/12665 | 1.09 | 0.92 | 1.31 | 4.07E-01 |
| POLICE OFFICERS (SERGEANT AND BELOW) | 3312 | 336/41453 | 109/12644 | 1.08 | 0.91 | 1.27 | 4.73E-01 |
| SALES REPRESENTATIVES | 3542 | 442/41347 | 140/12613 | 1.07 | 0.93 | 1.24 | 4.54E-01 |
| GARDENERS AND GROUNDSMEN/GROUNDSWOMEN | 5113 | 268/41521 | 95/12658 | 1.06 | 0.90 | 1.26 | 5.93E-01 |
| CIVIL SERVICE ADMINISTRATIVE OFFICERS AND ASSISTANTS | 4112 | 205/41584 | 75/12678 | 1.06 | 0.87 | 1.27 | 6.90E-01 |
| MARKETING AND SALES MANAGERS | 1132 | 1336/40453 | 403/12350 | 1.05 | 0.97 | 1.15 | 3.28E-01 |
| PRODUCTION, WORKS AND MAINTENANCE MANAGERS | 1121 | 1720/40069 | 487/12266 | 1.01 | 0.93 | 1.09 | 8.81E-01 |
| METAL WORKING MACHINE OPERATIVES | 8125 | 369/41420 | 116/12637 | 1.01 | 0.86 | 1.18 | 9.65E-01 |
| SECURITY GUARDS AND RELATED OCCUPATIONS | 9241 | 211/41578 | 76/12677 | 1.00 | 0.82 | 1.21 | 9.83E-01 |
| ENGINEERING TECHNICIANS | 3113 | 173/41616 | 54/12699 | 1.00 | 0.79 | 1.26 | 9.83E-01 |
| PROPERTY, HOUSING AND LAND MANAGERS | 1231 | 348/41441 | 103/12650 | 0.99 | 0.84 | 1.17 | 9.73E-01 |
| LOCAL GOVERNMENT CLERICAL OFFICERS AND ASSISTANTS | 4113 | 196/41593 | 59/12694 | 0.98 | 0.79 | 1.22 | 9.32E-01 |
| ELECTRICAL/ELECTRONICS ENGINEERS NEC | 5249 | 316/41473 | 94/12659 | 0.98 | 0.82 | 1.17 | 8.81E-01 |
| FINANCE AND INVESTMENT ANALYSTS/ADVISERS | 3534 | 383/41406 | 102/12651 | 0.97 | 0.81 | 1.15 | 7.92E-01 |
| BUS AND COACH DRIVERS | 8213 | 208/41581 | 72/12681 | 0.96 | 0.79 | 1.17 | 8.02E-01 |
| CIVIL SERVICE EXECUTIVE OFFICERS | 4111 | 221/41568 | 74/12679 | 0.96 | 0.79 | 1.18 | 8.00E-01 |
| ENGINEERING PROFESSIONALS NEC | 2129 | 294/41495 | 76/12677 | 0.95 | 0.78 | 1.16 | 7.35E-01 |
| QUANTITY SURVEYORS | 2433 | 199/41590 | 50/12703 | 0.95 | 0.74 | 1.22 | 7.92E-01 |
| SOLICITORS AND LAWYERS, JUDGES AND CORONERS | 2411 | 544/41245 | 129/12624 | 0.88 | 0.76 | 1.03 | 1.87E-01 |
| CHARTERED SURVEYORS (NOT QUANTITY SURVEYORS) | 2434 | 501/41288 | 113/12640 | 0.86 | 0.72 | 1.01 | 1.14E-01 |
| PERSONNEL, TRAINING AND INDUSTRIAL RELATIONS MANAGERS | 1135 | 296/41493 | 67/12686 | 0.85 | 0.68 | 1.05 | 1.87E-01 |
| MANAGEMENT CONSULTANTS, ACTUARIES, ECONOMISTS AND STATISTICIANS | 2423 | 695/41094 | 139/12614 | 0.80 | 0.68 | 0.93 | 7.93E-03 |
| CHARTERED AND CERTIFIED ACCOUNTANTS | 2421 | 589/41200 | 114/12639 | 0.79 | 0.67 | 0.94 | 1.50E-02 |
| VOCATIONAL AND INDUSTRIAL TRAINERS AND INSTRUCTORS | 3563 | 220/41569 | 51/12702 | 0.79 | 0.62 | 1.02 | 1.12E-01 |
| INFORMATION AND COMMUNICATION TECHNOLOGY MANAGERS | 1136 | 976/40813 | 201/12552 | 0.79 | 0.70 | 0.90 | 6.46E-04 |
| GENERAL OFFICE ASSISTANTS/CLERKS | 4150 | 392/41397 | 92/12661 | 0.79 | 0.66 | 0.95 | 2.40E-02 |
| FURTHER EDUCATION TEACHING PROFESSIONALS | 2312 | 356/41433 | 81/12672 | 0.78 | 0.64 | 0.95 | 2.54E-02 |
| SECONDARY EDUCATION TEACHING PROFESSIONALS | 2314 | 1076/40713 | 228/12525 | 0.78 | 0.69 | 0.88 | 1.51E-04 |
| PUBLIC SERVICE ASSOCIATE PROFESSIONALS | 3561 | 328/41461 | 68/12685 | 0.76 | 0.61 | 0.94 | 2.54E-02 |
| ACCOUNTS AND WAGES CLERKS, BOOK-KEEPERS, OTHER FINANCIAL CLERKS | 4122 | 708/41081 | 148/12605 | 0.76 | 0.66 | 0.88 | 6.27E-04 |
| MECHANICAL ENGINEERS | 2122 | 492/41297 | 93/12660 | 0.75 | 0.62 | 0.90 | 4.67E-03 |
| FINANCIAL INSTITUTION MANAGERS | 1151 | 401/41388 | 70/12683 | 0.71 | 0.57 | 0.88 | 5.51E-03 |
| SOFTWARE PROFESSIONALS | 2132 | 835/40954 | 163/12590 | 0.70 | 0.61 | 0.81 | 7.08E-06 |
| IT STRATEGY AND PLANNING PROFESSIONALS | 2131 | 691/41098 | 120/12633 | 0.69 | 0.59 | 0.82 | 5.27E-05 |
| CIVIL ENGINEERS | 2121 | 609/41180 | 89/12664 | 0.60 | 0.49 | 0.73 | 2.03E-06 |
| HIGHER EDUCATION TEACHING PROFESSIONALS | 2311 | 1127/40662 | 162/12591 | 0.55 | 0.47 | 0.63 | 7.62E-15 |
| ^NEC - Not Elsewhere Classified; NCO - non-commissioned officer^ |  |  |  |  |  |  |  |

**Table S8: Sensitivity analysis outcomes for females, ordered by PR**

| **Job** | **Job code** | **Controls in job / not in job** | **Controls in job / not in job** | **PR** | **LCL** | **UCL** | **P-FDR** |
| --- | --- | --- | --- | --- | --- | --- | --- |
| VOCATIONAL AND INDUSTRIAL TRAINERS AND INSTRUCTORS | 3563 | 233/40888 | 52/5102 | 1.61 | 1.26 | 2.06 | 2.21E-03 |
| PRODUCTION, WORKS AND MAINTENANCE MANAGERS | 1121 | 282/40839 | 56/5098 | 1.41 | 1.11 | 1.79 | 2.08E-02 |
| CARE ASSISTANTS AND HOME CARERS | 6115 | 610/40511 | 114/5040 | 1.36 | 1.15 | 1.60 | 3.45E-03 |
| MARKETING AND SALES MANAGERS | 1132 | 430/40691 | 83/5071 | 1.34 | 1.10 | 1.64 | 1.74E-02 |
| OFFICE MANAGERS | 1152 | 633/40488 | 111/5043 | 1.32 | 1.11 | 1.56 | 1.13E-02 |
| CIVIL SERVICE EXECUTIVE OFFICERS | 4111 | 383/40738 | 65/5089 | 1.22 | 0.98 | 1.53 | 2.18E-01 |
| CIVIL SERVICE ADMINISTRATIVE OFFICERS AND ASSISTANTS | 4112 | 418/40703 | 69/5085 | 1.21 | 0.97 | 1.50 | 2.27E-01 |
| PERSONNEL, TRAINING AND INDUSTRIAL RELATIONS MANAGERS | 1135 | 352/40769 | 54/5100 | 1.19 | 0.93 | 1.52 | 3.26E-01 |
| CLEANERS, DOMESTICS | 9233 | 388/40733 | 65/5089 | 1.17 | 0.94 | 1.46 | 3.26E-01 |
| HOUSING AND WELFARE OFFICERS | 3232 | 454/40667 | 67/5087 | 1.14 | 0.91 | 1.42 | 4.64E-01 |
| LOCAL GOVERNMENT CLERICAL OFFICERS AND ASSISTANTS | 4113 | 471/40650 | 69/5085 | 1.13 | 0.90 | 1.40 | 4.92E-01 |
| CUSTOMER CARE OCCUPATIONS | 7212 | 337/40784 | 51/5103 | 1.07 | 0.82 | 1.38 | 7.94E-01 |
| PUBLIC SERVICE ASSOCIATE PROFESSIONALS | 3561 | 411/40710 | 55/5099 | 1.05 | 0.82 | 1.34 | 8.48E-01 |
| PERSONAL ASSISTANTS AND OTHER SECRETARIES | 4215 | 1568/39553 | 180/4974 | 1.03 | 0.90 | 1.19 | 7.94E-01 |
| SALES AND RETAIL ASSISTANTS | 7111 | 978/40143 | 118/5036 | 1.01 | 0.85 | 1.19 | 9.71E-01 |
| FINANCIAL MANAGERS AND CHARTERED SECRETARIES | 1131 | 400/40721 | 50/5104 | 1.00 | 0.76 | 1.30 | 9.71E-01 |
| ACCOUNTS AND WAGES CLERKS, BOOK-KEEPERS, OTHER FINANCIAL CLERKS | 4122 | 1501/39620 | 179/4975 | 0.99 | 0.87 | 1.14 | 9.71E-01 |
| SOCIAL WORKERS | 2442 | 450/40671 | 57/5097 | 0.99 | 0.77 | 1.26 | 9.71E-01 |
| GENERAL OFFICE ASSISTANTS/CLERKS | 4150 | 2194/38927 | 246/4908 | 0.96 | 0.85 | 1.08 | 6.47E-01 |
| TEACHING PROFESSIONALS NEC | 2319 | 573/40548 | 60/5094 | 0.91 | 0.71 | 1.15 | 6.11E-01 |
| FURTHER EDUCATION TEACHING PROFESSIONALS | 2312 | 532/40589 | 57/5097 | 0.91 | 0.71 | 1.16 | 6.11E-01 |
| RECEPTIONISTS | 4216 | 736/40385 | 74/5080 | 0.90 | 0.73 | 1.12 | 5.65E-01 |
| SECONDARY EDUCATION TEACHING PROFESSIONALS | 2314 | 1602/39519 | 151/5003 | 0.84 | 0.72 | 0.98 | 8.18E-02 |
| HIGHER EDUCATION TEACHING PROFESSIONALS | 2311 | 729/40392 | 77/5077 | 0.84 | 0.68 | 1.04 | 2.43E-01 |
| NURSES | 3211 | 1962/39159 | 199/4955 | 0.82 | 0.72 | 0.94 | 1.74E-02 |
| EDUCATIONAL ASSISTANTS | 6124 | 996/40125 | 101/5053 | 0.77 | 0.64 | 0.93 | 2.28E-02 |
| PRIMARY AND NURSERY EDUCATION TEACHING PROFESSIONALS | 2315 | 2227/38894 | 136/5018 | 0.56 | 0.48 | 0.66 | 2.21E-10 |
| ^NEC - Not Elsewhere Classified; NCO - non-commissioned officer^ |  |  |  |  |  |  |  |

**Table 9**: Occupation categories associated with increased risk of heavy alcohol consumption in the entire cohort using two-digit SOC, V.2000.

| **Job** | **Code** | **Controls in job / not in job** | **Controls in job / not in job** | **PR** | **LCL** | **UCL** | **P-FDR** |
| --- | --- | --- | --- | --- | --- | --- | --- |
| SKILLED CONSTRUCTION AND BUILDING TRADES | 53 | 1570/81340 | 1066/16841 | 1.68 | 1.60 | 1.76 | 5.9E-98 |
| TEACHING AND RESEARCH PROFESSIONALS | 23 | 10539/72371 | 1252/16655 | 0.69 | 0.65 | 0.72 | 8.6E-42 |
| SCIENCE AND TECHNOLOGY PROFESSIONALS | 21 | 5065/77845 | 881/17026 | 0.71 | 0.67 | 0.76 | 6.1E-26 |
| MANAGERS AND PROPRIETORS IN AGRICULTURE AND SERVICES | 12 | 2255/80655 | 764/17143 | 1.38 | 1.30 | 1.46 | 6.1E-24 |
| SKILLED METAL AND ELECTRICAL TRADES | 52 | 2583/80327 | 1116/16791 | 1.28 | 1.22 | 1.35 | 3.7E-21 |
| HEALTH PROFESSIONALS | 22 | 1820/81090 | 158/17749 | 0.48 | 0.42 | 0.56 | 1.0E-20 |
| ELEMENTARY TRADES, PLANT AND STORAGE RELATED OCCUPATIONS | 91 | 857/82053 | 462/17445 | 1.39 | 1.30 | 1.50 | 1.7E-18 |
| BUSINESS AND PUBLIC SERVICE PROFESSIONALS | 24 | 6070/76840 | 1028/16879 | 0.78 | 0.73 | 0.82 | 9.1E-17 |
| PROCESS, PLANT AND MACHINE OPERATIVES | 81 | 1288/81622 | 568/17339 | 1.29 | 1.21 | 1.38 | 3.3E-13 |
| TRANSPORT AND MOBILE MACHINE DRIVERS AND OPERATIVES | 82 | 1624/81286 | 712/17195 | 1.22 | 1.14 | 1.30 | 2.3E-09 |
| ELEMENTARY ADMINISTRATION AND SERVICE OCCUPATIONS | 92 | 1960/80950 | 623/17284 | 1.21 | 1.13 | 1.30 | 5.5E-08 |
| HEALTH AND SOCIAL WELFARE ASSOCIATE PROFESSIONALS | 32 | 4652/78258 | 563/17344 | 0.81 | 0.75 | 0.87 | 2.2E-07 |
| LEISURE AND OTHER PERSONAL SERVICE OCCUPATIONS | 62 | 929/81981 | 272/17635 | 1.30 | 1.17 | 1.44 | 8.8E-07 |
| SCIENCE AND TECHNOLOGY ASSOCIATE PROFESSIONALS | 31 | 1494/81416 | 272/17635 | 0.77 | 0.69 | 0.86 | 5.9E-06 |
| CORPORATE MANAGERS | 11 | 12784/70126 | 3156/14751 | 1.07 | 1.03 | 1.11 | 3.0E-04 |
| ADMINISTRATIVE OCCUPATIONS | 41 | 8958/73952 | 1441/16466 | 0.93 | 0.88 | 0.97 | 4.6E-03 |
| TEXTILES, PRINTING AND OTHER SKILLED TRADES | 54 | 872/82038 | 265/17642 | 1.17 | 1.05 | 1.29 | 4.7E-03 |
| PROTECTIVE SERVICE OCCUPATIONS | 33 | 661/82249 | 222/17685 | 1.13 | 1.01 | 1.27 | 0.05 |
| SALES OCCUPATIONS | 71 | 1777/81133 | 356/17551 | 1.10 | 1.00 | 1.21 | 0.06 |
| SECRETARIAL AND RELATED OCCUPATIONS | 42 | 3534/79376 | 391/17516 | 0.92 | 0.83 | 1.01 | 0.10 |
| CUSTOMER SERVICE OCCUPATIONS | 72 | 559/82351 | 102/17805 | 0.91 | 0.76 | 1.09 | 0.37 |
| CULTURE, MEDIA AND SPORTS OCCUPATIONS | 34 | 2416/80494 | 511/17396 | 0.97 | 0.90 | 1.05 | 0.55 |
| CARING PERSONAL SERVICE OCCUPATIONS | 61 | 2977/79933 | 466/17441 | 0.99 | 0.91 | 1.08 | 0.87 |
| BUSINESS AND PUBLIC SERVICE ASSOCIATE PROFESSIONALS | 35 | 5101/77809 | 1112/16795 | 1.00 | 0.95 | 1.06 | 0.99 |
| SKILLED AGRICULTURAL TRADES | 51 | 565/82345 | 148/17759 | 1.00 | 0.87 | 1.15 | 0.99 |

**Table S10: Comparison of the frequency (%) of major occupation groups between the Labour Force Survey and the subset of UK Biobank used for study analysis**

|  | **Labour Force survey 2008 (n = 29,475,000^a^)**  **No. (%)** | **UK Biobank subset (n = 100,817)**  **No. (%)** |
| --- | --- | --- |
|  |  |  |
| **Managers and Senior Officials** | 4,590,000 (15.6) | 18,959 (18.8) |
| **Professional occupations** | 3,776,000 (12.8) | 26,813 (26.6) |
| **Associate Professional and Technical** | 4,302,000 (14.6) | 17,004 (16.9) |
| **Administrative and Secretarial** | 3,340,000 (11.3) | 14,324 (14.2) |
| **Skilled Trades Occupations** | 3,221,000 (10.9) | 8,185 (8.1) |
| **Personal Service Occupations** | 2,432,000 (8.3) | 4,644 (4.6) |
| **Sales and Customer Service Occupations** | 2,228,000 (7.6) | 2,794 (2.8) |
| **Process Plant and Machine Operatives** | 2,083,000 (7.1) | 4,192 (4.2) |
| **Elementary Occupations** | 3,411,000 (11.6) | 3,902 (3.9) |

^a.^ Raw data presented as per thousand (‘000). Carry-over effect means percent total does not equal 100.

**Table S11. Comparison of the proportion of major occupation groups between the Labour Force Survey 2008 and 2018**

|  | **Labour Force survey 2008. %** | **Labour Force survey 2018. %** |
| --- | --- | --- |
| **Managers and Senior Officials** | 15.6 | 10.8 |
| **Professional occupations** | 12.8 | 20.1 |
| **Associate Professional and Technical** | 14.6 | 14.5 |
| **Administrative and Secretarial** | 11.3 | 10.5 |
| **Skilled Trades Occupations** | 10.9 | 10.2 |
| **Personal Service Occupations** | 8.3 | 9.2 |
| **Sales and Customer Service Occupations** | 7.6 | 7.6 |
| **Process Plant and Machine Operatives** | 7.1 | 6.4 |
| **Elementary Occupations** | 11.6 | 10.7 |
